# Genetic structure and population connectivity of the blue and red shrimp *Aristeus antennatus*

**DOI:** 10.1038/s41598-019-49958-5

**Published:** 2019-09-19

**Authors:** Sandra Heras, Laia Planella, José-Luis García-Marín, Manuel Vera, María Inés Roldán

**Affiliations:** 1 Laboratori d’Ictiologia Genètica, Universitat de Girona, Girona, Spain; 2 Present Address: Departamento de Zoología, Genética y Antropología Física, Campus Lugo, Universidade de Santiago de Compostela, Lugo, Spain

**Keywords:** Genetic variation, Zoology

## Abstract

The blue and red shrimp *Aristeus antennatus* is a demersal marine species harvested by bottom trawling in the Mediterranean Sea, the adjacent Atlantic Ocean (AO) waters, and the Mozambique Channel in the Indian Ocean (IO). As it is considered to be a priority species for sustainable fishing, identification of its genetic stocks and the connectivity between them is essential. Using 12 microsatellite loci we detected at least four genetic stocks distributed in the Western Mediterranean (WM), Eastern Mediterranean (EM), AO, and IO and signals for a possible fifth stock in the Alborán Sea. We detected no additional population structuring within the WM. Thus, although the Almería-Orán Front exerts some isolating effect, high genetic homogeneity and gene flow are present within the WM Basin. The IO stock is genetically closer to the AO stock than to the others; thus, the species dispersion route is more likely via the Atlantic Ocean than via the Red Sea. Large effective population sizes suggest population sustainability, but moderate genetic diversity values indicate to proceed with caution. Our genetic results serve as a basis for species conservation to ensure long-term sustainability of this marine resource.

## Introduction

The blue and red shrimp *Aristeus antennatus* (Risso, 1816) (Penaeoidea, Aristeidae) is a demersal marine decapod that inhabits the muddy bottoms of the continental slope along submarine canyons^1^. The species is distributed throughout the Mediterranean Sea, in the Atlantic Ocean (AO) waters adjacent to the Strait of Gibraltar from Portugal to Cape Verde, and in the western Indian Ocean (IO) from South Africa across the Mozambique Channel, Zanzibar, and the Maldives Islands; more recently, its presence off the coast of Brazil has been suggested^2^. Biological studies indicate that this species is the most eurybathic in the Mediterranean Sea, present at depths ranging from 80 to 3,300 m, with a greater abundance in the first 1,000 m depth, where fishing occurs^3^.

*A. antennatus* is among the most important demersal resources for bottom-trawling fishery in the Mediterranean Sea, with 1,782 tonnes collected in 2014^4^. In fact, since 2006, the General Fisheries Commission for the Mediterranean Sea included *A. antennatus* on the list of priority species for which fishing regulation plans should be developed^5^. It is highly appreciated gastronomically and retail market prices are quite high ($70–160/kg) in the Western Mediterranean (WM), where it has been fished most intensely. Outside the Mediterranean Sea, other fishing zones of lesser importance extend to the south of Portugal^6^, and a new fishing ground was established recently in the Mozambique Channel^7^.

However, no sustainable fishing management policy for *A. antennatus* exists at the international level yet. Long-term species sustainability requires adequate fishing and conservation strategies that depend heavily on the identification of the species’ population structure. One of the primary tools for this purpose is genetic analysis, which involves the identification of genetic stocks and connectivity among them^8^. Such analysis is particularly important to study population processes of marine organisms from inaccessible habitats, such as deep-sea species^9^.

A previous study using two mitochondrial DNA (mtDNA) markers (16S rDNA and COI) detected four genetic stocks of *A. antennatus* in the range of distribution where the species is exploited: the AO, WM, Eastern Mediterranean (EM), and IO^2^. However, no mtDNA marker study to date has been able to discern genetic structuring at a more regional level between proximal fishing zones^2,10–12^.

A priori, the examination of more variable molecular markers, such as microsatellites, which are sensitive to small but significant genetic differences, should reveal genetic structure at a finer regional scale in populations of *A. antennatus* and the degree of connectivity between stocks. Microsatellite analysis has enabled the identification of stocks in other penaeoid marine shrimp species (reviewed in Heras *et al*.^13^). However, very few microsatellite markers are available for *A. antennatus* (14 are reported in Cannas *et al*.^14^), and they have not been proven to be useful for the detection of genetic stocks^15^. This situation led us to develop and characterise 35 new microsatellite loci for *A. antennatus* using next-generation sequencing^13^ and in the present study we used 12 of them.

**Figure 1:**
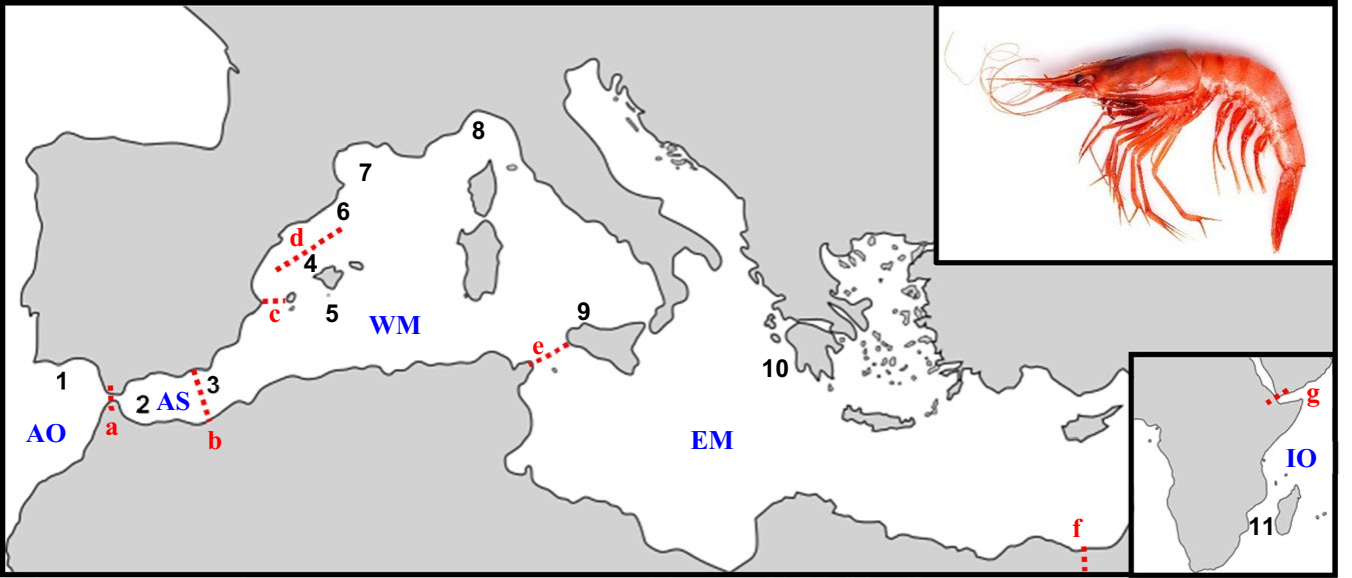
Main geographic and oceanographic barriers in the Mediterranean Sea and Red Sea. Sampling locations of *A. antennatus* in the five analysed regions: Atlantic Ocean (AO): 1 Faro; Alborán Sea (AS): 2 Alborán Sea; Western Mediterranean (WM): 3 Almería, 4 Sóller, 5 Cabrera, 6 Palamós, 7 Gulf of Lion, 8 Genoa, 9 Palermo; Eastern Mediterranean (EM): 10 Ionian Sea; Indian Ocean (IO): 11 Mozambique Channel. Main geographic and oceanographic barriers in the Mediterranean Sea and Red Sea are represented with dashed lines: (**a**) Strait of Gibraltar, (**b**) Almería-Orán Front, (**c**) Ibiza Channel, (**d**) Balearic Front, (**e**) Strait of Sicily, (**f**) Suez Canal, (**g**) Strait of Bab-el-Mandeb.

Although human impacts, particularly through fishing^16^, affect the population configuration of marine organisms, other factors such as marine geology and ocean currents create many geographic and oceanographic discontinuities that can reduce connectivity, and strongly influence population structure affecting effective species conservation strategies, as has been observed in numerous Mediterranean species (reviewed by Patarnello *et al*.^17^ and Pascual *et al*.^18^). In the Mediterranean Sea, they include the following (Fig. 1):

(i) The Strait of Gibraltar, which forms the dividing zone between the denser Mediterranean waters and the Atlantic Ocean waters.
(ii) The Almería-Orán Front, formed at the eastern end of the anticyclonic Eastern Alborán Gyre, between the coasts of the Iberian Peninsula and Algeria.
(iii) The Balearic Front and Ibiza Channel, which act in the area between the Balearic Islands and the Iberian Peninsula.
(iv) The Strait of Sicily separating the Western Mediterranean Basin from the Eastern Mediterranean Basin.

On the other hand, the Red Sea is a possible connection between the Indian Ocean and the Mediterranean Sea, but the Bab-el-Mandeb Strait and the Suez Canal are possible barriers along this route (Fig. 1).

By using genetic variation at 12 microsatellite loci, the aim of this study is the identification of genetic stocks across the distribution of *A. antennatus* and the effect of putative geographic and oceanographic barriers, indicated before, to prevent connectivity among stocks.

## Results

### Genetic diversity and effective population size

The 12 microsatellite loci were polymorphic at all locations analysed and presented a total of 158 alleles, with an average of 13.2 (range, 4–40) alleles per locus, of which 19% (*n* = 30) were private alleles present at single locations. Among five sampled regions, Mozambique contributed the largest number of alleles (Ap = 17; Table 1). Genotype linkage disequilibrium was not detected in any pair of loci across the 11 sampled locations.

**Table 1:** Genetic diversity and effective population size in eleven *Aristeus antennatus* locations.

Region and Sample Location	Code	*N*	*N*a	Ar	Ap	*H*o	*H*e	*F*_IS_	*N*_*e*_	CI for *N*_*e*_
Atlantic Ocean	AO									
Faro, Portugal	Fa	38	7.6	7.6	1	0.435	0.628	0.307	241.0	88.0–infinite
Alborán Sea	AS									
Alborán Sea, Spain	Alb	53	8.0	7.4	0	0.413	0.620	0.333	21,464.7	198.5–infinite
Western Mediterranean	WM									
Almería, Spain	Al	45	7.3	7.1	1	0.480	0.624	0.231	Infinite	131.9–infinite
Sóller, Spain	So	47	8.0	7.5	3	0.466	0.641	0.273	Infinite	163.9–infinite
Cabrera, Spain	Ca	40	7.4	7.3	1	0.496	0.632	0.216	4,121.8	147.8–infinite
Palamós, Spain	Pa	49	7.3	6.9	1	0.406	0.603	0.327	Infinite	992.8–infinite
Gulf of Lion, France	GL	50	8.3	7.7	3	0.442	0.619	0.286	Infinite	474.9–infinite
Genoa, Italy	Ge	44	7.5	7.2	0	0.491	0.629	0.220	Infinite	285.7–infinite
Palermo, Italy	Po	40	7.6	7.5	2	0.442	0.614	0.277	Infinite	108.0–infinite
Eastern Mediterranean	EM									
Ionian Sea, Greece	IS	40	7.1	7.0	1	0.444	0.613	0.276	Infinite	156.4–infinite
Indian Ocean	IO									
Mozambique Channel, Mozambique	Moz	48	9.8	9.1	17	0.522	0.684	0.237	Infinite	Infinite–infinite
Total/average		494	7.8	7.5	2.7	0.458	0.628	0.271	Infinite	Infinite–infinite

*N*, sample size; *N*a, number of alleles per locus; Ar, allelic richness per locus (based on minimum sample size of 37 diploid individuals with complete genotypes); Ap, number of private alleles; *H*o, observed heterozygosity; *H*e, expected heterozygosity; *F*_IS_, inbreeding coefficient; *N*_*e*_, effective population size; CI, confidence interval (jack-knife method).

All individuals displayed ≥11 genotyped loci.

Analysis of variability and genetic diversity by location yielded moderate values, with an average of 7.8 (range, 7.1–9.8) alleles per locus, average allelic richness (Ar) value of 7.5 (range, 6.9–9.1), and mean observed and expected heterozygosities (*H*o and *H*e, respectively) of 0.458 (range, 0.406–0.522) and 0.628 (range, 0.603–0.684), respectively. Mozambique sample presented the highest values in all cases (Table 1).

Among the 12 microsatellite loci only Aa496b and Aa1195 complied with HW equilibrium genotype proportions at all locations (Supplementary Table S1). On average, all locations showed deviation from Hardy-Weinberg (HW) equilibrium related with positive *F*_IS_ values, suggesting a deficit of heterozygotes (Table 1). The observed heterozygote deficit might be due to the presence of null alleles. With the exception of the Aa1195 locus, Micro-Checker analysis suggested the presence of null alleles at the other loci in at least one sample (with a maximum allele frequency of 0.2893 at Aa818 locus in Palermo), but never the same loci across locations. Given the lack of consistency among locations in the presence of null alleles, all loci were included in further analysis.

The estimates of effective population size (*N*_*e*_) using NeEstimator software often yielded negative values that were interpreted as very large (infinite; Table 1)^19^. Finite values were obtained only for Faro, Alborán, and Cabrera locations (241.0, 21,464.7 and 4,121.8, respectively), although even in these cases the upper boundaries of the 95% confidence intervals were infinite (Table 1).

### Population structure and gene flow

**Figure 2:**
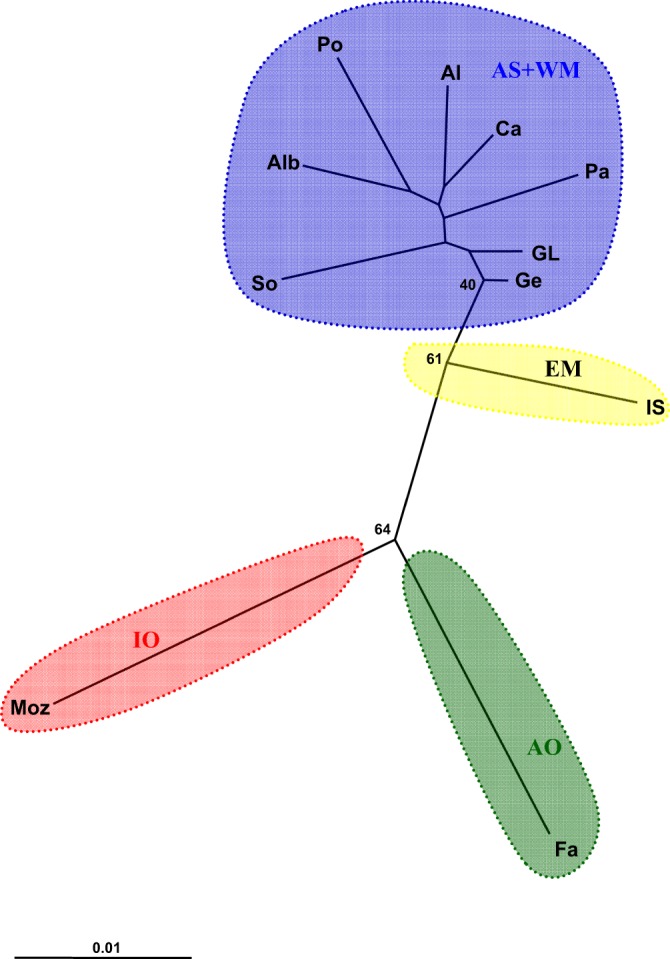
Neighbour-joining tree based on Latter’s *F*_ST_ genetic distance^56^. ≥40% bootstrap values based on 1,000 replicates are indicated next to nodes. Region and location codes are given in Table 1.

Pairwise *F*_ST_ between all locations in the WM were very low (average, 0.00394) and not significant (Table 2). In addition, the AS was not significantly differentiated from the WM locations, indicative of a single population in the Western Mediterranean Sea (WM + AS). Comparative values between the IO and AO regions, respectively, and the other regions were highest (averages, 0.03609 and 0.03170, respectively) and statistically significant. The EM sample exhibited marked and significant differentiation compared with the AO (0.02413) and IO (0.03578), and less differentiation with the WM (average, 0.01526) and Alborán Sea (AS; 0.01261) locations, with significant values obtained only for the Palamós and Palermo samples. In addition, the neighbour-joining (NJ) tree summarised the geographical population groups (Fig. 2) suggested by pairwise *F*_ST_ distance analysis.

**Table 2:** Pairwise *F*_ST_ values between the 11 sampling sites (below the diagonal) and *p* values (above the diagonal).

		AO	AS	WM							EM	IO
		Fa	Alb	Al	So	Ca	Pa	GL	Ge	Po	IS	Moz
AO	**Fa**	—	**0.00000**	**0.00000**	**0.00000**	**0.00000**	**0.00000**	**0.00010**	**0.00010**	**0.00000**	**0.00030**	**0.00000**
AS	**Alb**	0.03396	—	0.34175	0.03782	0.38452	0.07029	0.35412	0.76091	0.49342	0.01049	**0.00000**
WM	**Al**	0.03448	0.00394	—	0.02841	0.94119	0.33482	0.23760	0.29621	0.34719	0.00168	**0.00000**
	**So**	0.04191	0.00888	0.00963	—	0.49134	0.08069	0.30611	0.28859	0.01485	0.00396	**0.00000**
	**Ca**	0.03513	0.00372	0.00000	0.00268	—	0.50292	0.67617	0.78764	0.49797	0.00446	**0.00000**
	**Pa**	0.03534	0.00814	0.00410	0.00808	0.00270	—	0.77507	0.27067	0.19454	**0.00030**	**0.00000**
	**GL**	0.02521	0.00392	0.00475	0.00422	0.00106	0.00044	—	0.99119	0.26453	0.07643	**0.00000**
	**Ge**	0.02278	0.00061	0.00392	0.00433	0.00000	0.00480	0.00000	—	0.57034	0.23760	**0.00000**
	**Po**	0.03515	0.00314	0.00399	0.01191	0.00271	0.00633	0.00509	0.00200	—	**0.00010**	**0.00000**
EM	**IS**	0.02413	0.01261	0.01582	0.01517	0.01553	0.02158	0.00899	0.00531	0.02443	—	**0.00000**
IO	**Moz**	0.02892	0.04379	0.03887	0.02910	0.03107	0.04346	0.03296	0.02997	0.04695	0.03578	—

Region and location codes are given in Table 1. Significance after Bonferroni correction indicated in bold (*p* < 0.00091).

We confirmed that the frequency of null alleles did not alter our results, as the pairwise *F*_ST_ values were similar with and without correction for the presence of null alleles (Supplementary Table S2). Thus, the effect of null alleles was negligible when estimating population genetic differentiation.

Analysis of molecular variance (AMOVA) revealed significant genetic structuring in all hypothesised grouping scenarios, with significant *F*_ST_ values due primarily to differences among groups (*F*_CT_), rather than within groups (*F*_SC_). The first and fourth hypotheses yielded the most significant *F*_CT_ values and no significant divergence within groups (Table 3). For the fourth hypothesis (genetic structuring in five groups), the Almería-Orán Front was added to the barriers included in the first one. Similar results were obtained in both analyses, suggesting that genetic structuring in five groups is as plausible as that in four groups, with the Almería-Orán Front possibly contributing to isolate the *A. antennatus* population in the AS from the WM ones. Compared with the first and fourth hypotheses, which included the Strait of Gibraltar, the second one, in which the Strait of Gibraltar did not act as barrier, showed increased, significant variance among samples within groups (*F*_SC_ = 0.00898, *p* < 0.00001) due to the low homogeneity within the groups created (Table 3). These results supported the hypothesis that the Strait of Gibraltar acts as an effective barrier for *A. antennatus*. Similarly, comparison of the third hypothesis with the most plausible first and fourth hypotheses suggested that the Strait of Sicily also acts as an oceanographic barrier for this species (*F*_SC_ = 0.00623, *p* = 0.00713; Table 3).

**Table 3:** Hierarchical analysis of molecular variance (AMOVA) based on different grouping hypotheses.

Hypothesis	Variance	% Variation	*F* statistics
1. **Four groups** (mtDNA: Strait of Gibraltar and Strait of Sicily acting) **AO, AS** + **WM, EM, IO**			
Among groups	0.09986	2.58	*F*_CT_ = 0.02577***
Among samples within groups	0.01524	0.39	*F*_SC_ = 0.00404
Within samples	3.75983	97.03	*F*_ST_ = 0.02970***
2. **Four groups** (mtDNA; +Almería-Orán Front, Strait of Gibraltar not acting) **AO** + **AS, WM, EM, IO**			
Among groups	0.04631	1.20	*F*_CT_ = 0.01206**
Among samples within groups	0.03406	0.89	*F*_SC_ = 0.00898***
Within samples	3.75983	97.91	*F*_ST_ = 0.01206***
3. **Four groups** (mtDNA; Strait of Sicily not acting) **AO, AS** + **WM, Po** + **EM, IO**			
Among groups	0.06708	1.74	*F*_CT_ = 0.01742***
Among samples within groups	0.02356	0.61	*F*_SC_ = 0.00623**
Within samples	3.75983	97.65	*F*_ST_ = 0.02354***
4. **Five groups** (mtDNA; +Almería-Orán Front) **AO, AS, WM, EM, IO**			
Among groups	0.07711	2.00	*F*_CT_ = 0.02002***
Among samples within groups	0.01421	0.37	*F*_SC_ = 0.00377
Within samples	3.75983	97.63	*F*_ST_ = 0.02371***

Region and location codes are given in Table 1. MtDNA (four groups based on previous mitochondrial DNA study by Fernández *et al*.^10^). **p < 0.01, ***p < 0.001.

**Figure 3:**
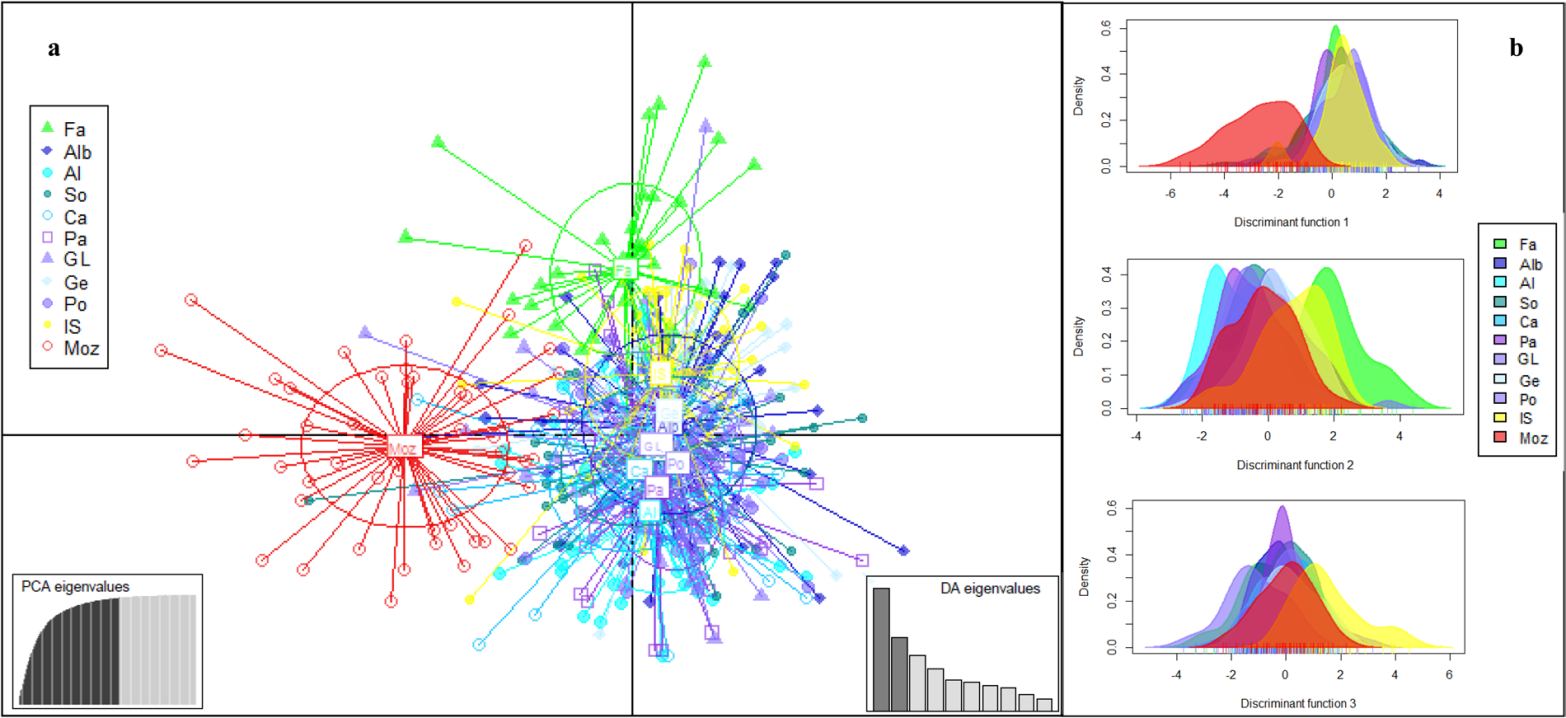
Discriminant analysis of principal components (DAPC) results. **(a)** Scatterplot showing the first two principal components from DAPC. Individuals are represented as symbols and groups derived from locations as inertia ellipses. The eigenvalues retained in principal component analysis (PCA) and discriminant analysis (DA) are indicated. (**b)** Individual density plots for the three first discriminant functions. Each colour represents a location. Location codes are given in Table 1.

Discriminant analysis of principal components (DAPC) enabled differentiation of four groups: IO (Mozambique), AO (Faro), EM (Ionian Sea), and AS + WM (Fig. 3). The horizontal scatterplot axis, explaining 28.54% of variance, differentiated individuals from Mozambique; the vertical axis, explaining 17.25% of variance, differentiated individuals from Faro; and the third axis, explaining 12.94% of variance, differentiated individuals from the Ionian Sea. The proportion of individuals assigned to locations as inferred from membership probabilities was largest for the location of origin in all cases (Table 4). The highest values were for Mozambique (0.7708) and Faro (0.7368), which showed the least admixture, followed by the Ionian Sea (0.6250), with some admixture, primarily with individuals from AS and WM (Table 4, Supplementary Fig. S3). The AS and WM samples had lower proportions of assignment to their original location, but in general the second highest percentage of assignment was for another location in the same cluster, suggesting admixture between AS and WM populations.

**Table 4:** Proportions of individuals assigned to their original locations (in bold) and to other locations.

Original location	Assigned location										
	Fa	Alb	Al	So	Ca	Pa	GL	Ge	Po	IS	Moz
Fa	**0.7368**	0.1315	0.0000	0.0000	0.0000	0.0263	0.0000	0.0263	0.0263	0.0263	0.0263
Alb	0.0188	**0.5471**	0.0188	0.0000	0.0566	0.0377	0.0377	0.0566	0.1132	0.0750	0.0377
Al	0.0444	0.0222	**0.5330**	0.0666	0.0666	0.1110	0.0222	0.0222	0.0444	0.0444	0.0222
So	0.0000	0.0850	0.0638	**0.5106**	0.0638	0.1063	0.0212	0.0638	0.0426	0.0212	0.0212
Ca	0.0000	0.0250	0.1000	0.0500	**0.3500**	0.0750	0.0750	0.1000	0.1000	0.0500	0.0750
Pa	0.0612	0.0204	0.0816	0.0204	0.0408	**0.5102**	0.0612	0.0408	0.0612	0.0612	0.0204
GL	0.0800	0.0400	0.0200	0.0600	0.0200	0.0600	**0.5200**	0.0600	0.0600	0.0400	0.0400
Ge	0.0227	0.1590	0.0227	0.0455	0.0227	0.1590	0.0227	**0.3181**	0.0909	0.1136	0.0227
Po	0.0750	0.1500	0.0250	0.0500	0.0500	0.0500	0.0750	0.0000	**0.4500**	0.0750	0.0000
IS	0.0250	0.0000	0.0750	0.0500	0.0250	0.0000	0.0500	0.1000	0.0000	**0.6250**	0.0500
Moz	0.0625	0.0417	0.0417	0.0000	0.0000	0.0208	0.0208	0.0000	0.0000	0.0417	**0.7708**

Location codes are given in Table 1.

We detected significant correlation between genetic and geographical distances for both: the Atlantic route (*r* = 0.67579, *p* = 0.00842) and the Red Sea route (*r* = 0.6502, *p* = 0.00783; Supplementary Fig. S4). The correlation still held when Mozambique, the most distant sample, was excluded from the analysis (*r* = 0.31811, *p* = 0.03937; Supplementary Fig. S4). However, when we eliminated Mozambique and EM, the second most distant sample located beyond the possible oceanographic barrier of the Strait of Sicily, the result was not significant (*r* = 0.3257, *p* = 0.07339). When we excluded Mozambique and Faro (AO), which is located on the other side of the Strait of Gibraltar, but retained the EM sample, again a significant correlation was obtained (*r* = 0.4057, *p* = 0.02401), indicating a pattern of isolation by distance (IBD) along the Mediterranean Sea.

**Figure 4:**
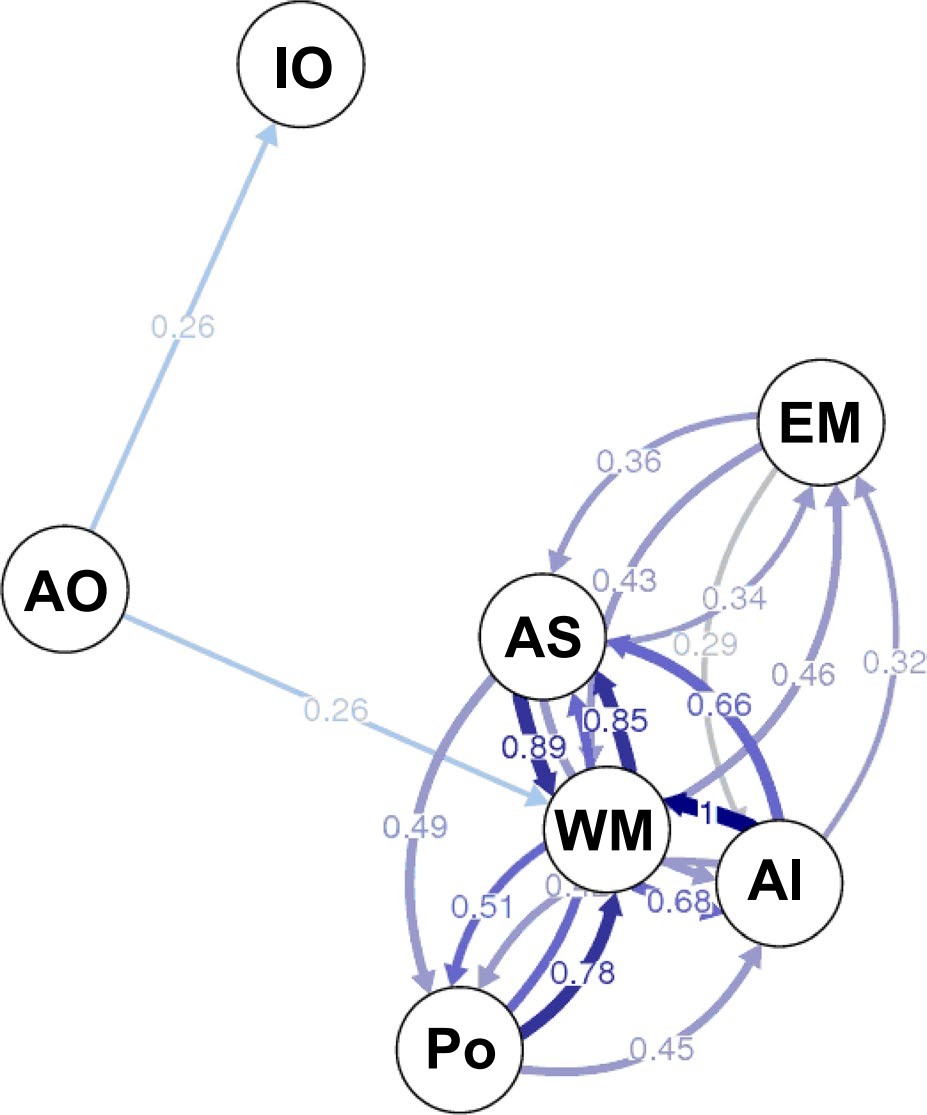
Relative migration network created using *Nm*^61^. Five groups hypothesis (Table 3) is considered and Palermo and Almería samples, which are adjacent to the tested oceanographic and geographic discontinuities, were also included. The proximity of nodes indicates more gene flow among them than with others, and the widths of arrows are proportional to relative migration values (threshold = 0.25). For WM, all WM samples except those from Palermo and Almería were pooled. Region and location codes are given in Table 1.

The migration network showed the greatest relative gene flow among WM Basin nodes (WM, AS, Palermo, and Almería) (Fig. 4), which agreed with the lower genetic differentiation observed among these locations (Table 2). The EM node was closest to the other Mediterranean nodes, with no relevant gene flow with the IO node. The AO and IO were the most distant regions (with the AO closer to the Mediterranean than the IO), as indicated by their lower relative rates of migration. We found no significant evidence of asymmetric gene flow between nodes.

## Discussion

Our analysis yielded higher average Na values (7.8; Table 1) than reported previously for the same loci by Heras *et al*.^13^, likely because of higher sample size in the present study. Our overall median *H*o and *H*e values (0.458 and 0.628; Table 1) were in the range of other reports for *A. antennatus*^13,14^ and the related *Aristaeomorpha foliacea* (Aristeidae)^20^. The Mozambique sample presented the highest levels of diversity (*N*a, Ar, *H*o, and *H*e in Table 1), followed by the Atlantic sample and then the Mediterranean ones, which suggests that the origin of *A. antennatus* is outside the Mediterranean Sea. The majority of species currently found in the Mediterranean Sea are likely the result of colonisation from the Atlantic Ocean, due to the tectonic opening of the Strait of Gibraltar after 0.5 million years of the Messinian Salinity Crisis (5.9–5.3 Ma), and to a much lesser degree from the Red Sea^17,21^.

The heterozygote deficit, reflected in positive *F*_IS_ values (average = 0.271; Table 1), was responsible for the observed HW deviations. We ruled out possible inbreeding, as a recent study suggested that spawning groups of this species are formed mostly by unrelated individuals^22^. We believe that this deficit may be due to the null alleles as previously observed for *A. antennatus* and other decapods^13,15^ and in the case of WM, they could also be due to the Wahlund effect.

The *N*_*e*_ is an indicator of population viability and should not fall below 50, as was observed in our study (Table 1), to avoid population extinction from inbreeding depression^23^. Thus, although *A. antennatus* is subject to intensive fishing, which is lower in Mozambique as suggested by the very large (infinite) lower limit of CI, the risk of demographic reduction due to inbreeding is low. Nevertheless, as estimates of genetic diversity at our study were not particularly high, caution is warranted because overfishing could result in the loss of essential alleles for adaptation to environmental changes^16^.

The *F*_ST_ values obtained in our study may appear to be low, but they were significant among the principal groups detected (Table 2). Low *F*_ST_ values, even those lower than 0.01, are common in marine species^24^, and high *N*_*e*_, as detected in our study, can lead to low degrees of genetic differentiation in neutral markers such as microsatellites^25^. A study on decapod crustaceans suggested that species inhabiting deeper waters, such as *A. antennatus*, present lower genetic differentiation than those in shallower waters^26^. We detected at least four genetic stocks across the distribution of *A. antennatus*, consisting of the AO, WM, EM, and IO stocks (Tables 2, 3, Figs 2, 3). This result agrees with the previously reported genetic structure of the species from mtDNA variation^2^. However, with the use of more sensitive markers, we revealed a possible new fifth genetic stock, the AS (Table 3).

A recent study showed that Mediterranean marine species, including crustaceans, with moderate or elevated dispersal capacity [planktonic larval duration (PLD) >2 weeks] are significantly affected by oceanographic discontinuities that reduce gene flow between populations^18^. The estimated PLD of *A. foliacea* could exceed 3 weeks^27^, thus, through extrapolation to *A. antennatus*, whose larval duration is still unknown, we can infer that the species likewise would be affected by such discontinuities.

The observed genetic differentiation of *A. antennatus* between the AO and Mediterranean Sea (Table 2, Figs 2, 3, 4) fits the phylogeographic pattern detected in the majority of Mediterranean marine species, including decapods^17,28–31^. According to a review^18^, the Strait of Gibraltar is the present-day biogeographic barrier that acts most strongly to reduce gene flow between North Atlantic and Mediterranean populations, also affecting decapods^26^. Based on our findings, it is the primary discontinuity marking the Atlantic–Mediterranean transition for *A. antennatus*, as detected previously with mtDNA analyses^2,12^. These results differ from the conclusions of Lo Brutto *et al*.^32^ using mtDNA and amplified fragment length polymorphisms (AFLPs) in *A. antennatus*, as they reported no significant genetic variation between the Atlantic and the Mediterranean populations. Considering that the depth of maximum abundance of *A. antennatus* adults (600–800 m)^3^ falls below the maximum sill depth in the Strait of Gibraltar (284 m at a width of ~30 km)^21^, the dispersal of adults is restricted, although possible planktonic larval drift by surface currents would not^33–36^.

Previous mtDNA analyses failed to identify the Almería-Orán Front as a barrier between populations of *A. antennatus*^2^. However, our results indicated a slight isolation of the AS sample from the other WM samples (Table 3), despite the gene flow suggested between them (Fig. 4). The size, position, and intensity of this front are variable and depend primarily on the Eastern Alborán Gyre, which collapses and gradually regenerates with a periodicity of 4 weeks^37^. Therefore, the semi-permanent and variable nature of the front helps to explain the tenuous effect we observed. The mtDNA analyses have shown that the Almería-Orán Front affects some crustacean species, such as *Meganyctiphanes norvegica* (euphasid) and *Palaemon elegans* (decapod)^38,39^, although it seems to cause no effect on other marine decapod species, including *Perapenaeus longirostris* (Penaoidea)^26^.

Although the Ibiza Channel and the Balearic front affected the decapod *Liocarcinus depurator*^26^ and littoral fish^40^, respectively, they had no effect on *A. antennatus* genetic structuring, confirming the results obtained using mtDNA^2,10–12^ and other microsatellite loci^15^. The detection of several larval stages of *A. antennatus* in this area^34–36^ reinforce the hypothesis that larvae are passively transported by oceanic currents in most of the marine decapods^33^, and justify the observed high degree at gene flow in the Western Mediterranean (Fig. 4, Table 4, Supplementary Fig. S3). The surface Northern Current and the circulation of deeper waters in the great cyclonic gyres of the WM, such as the Lion Gyre, could contribute to the horizontal and vertical population admixture in the WM Basin^21,41^. Moreover, periodical cascading events in the Gulf of Lion propagate shelf waters in submarine canyons at depths >2,000 m^1^.

The Strait of Sicily acts as a weak geographic barrier to gene flow in many marine species^18^. Available studies using mitochondrial or microsatellite DNA markers suggested that this barrier limits gene flow between the EM and WM in the decapods *Palinurus elephas* and *Crangon crangon*^28,29,31^. In contrast, no differentiation was observed in *Melicertus* (*Penaeus*) *kerathurus*^30^. Our analysis showed genetic differentiation between the WM and EM (Table 2, Fig. 2), consistent with the mtDNA results reported for *A. antennatus* by Fernández *et al*.^2^. Nevertheless, in other mtDNA studies, with samples geographically closer to both sides of the Strait of Sicily, no differentiation in *A. antennatus* between the two Mediterranean basins was detected^10,12^. This observation is consistent with the IBD pattern that we observed in the Mediterranean Sea (Supplementary Fig. S4) and suggests that the Strait of Sicily is a more permeable barrier than the Strait of Gibraltar for *A. antennatus*, given that the former is much deeper (360–430 m) and wider (130 km) than the latter^21^. IBD signals along the WM–EM axis also have been detected in the decapod *P. longirostris*^42^. Thus, the EM stock of *A. antennatus* might be affected by adaptations to the Eastern Mediterranean Basin due to differences in environmental conditions from the Western Mediterranean Basin^21^.

The IO stock presented the greatest genetic distance (Table 2) and was the most geographically isolated (Fig. 1). Although more than 33 species of decapods, including penaeoid shrimps, display Lessepsian migration from Indian Ocean to the Mediterranean Sea^43,44^, our findings indicate that the IO stock of *A. antennatus* is genetically closer to the AO stock than to the Mediterranean ones (Table 2, Figs 2, 4). These results suggest that the Suez Canal, with only 26 m depth, the Bab-el-Mandeb Strait, 137 m deep in the shallowest section^45^, and the Red Sea, with lesser average depth than the Mediterranean (450 m vs. 1,500 m)^21,45^, prevent connectivity between the Mediterranean Sea and Indian Ocean populations, and the dispersal route of *A. antennatus* is more likely via the Atlantic Ocean than via the Red Sea. On the other hand, an open question is raised, applicable also to other demersal species for which little information is available, of how we may explain the scant presence of *A. antennatus* along the Atlantic Coast of Africa^2^. The species may have a “patchwork” distribution, or demersal populations may remain undiscovered.

In conclusion, using a set of sensitive microsatellite loci in *A. antennatus*, we were able to detect genetic differentiation and identify at least four genetic stocks and the signal for a fifth stock along the species’ distribution. Our results support efforts to achieve sustainability of this resource for future generations via genetic assessment. Currently, overfishing strongly affects marine natural resources^16^. Taking into account genetic connectivity are fundamentally important to provide a basis for the study of *A. antennatus* resilience and adaptation to possible environmental changes from human impact^46^.

## Materials and Methods

### Sample sites

Samples were collected from 11 locations where *A. antennatus* is currently exploited across the following five regions: AO, AS, WM, EM, and IO. As the species is traditionally harvested in waters of the WM^4^, more locations from this region were included in the sampling design (Fig. 1, Table 1). In 2008, samples from AS, Almería, Sóller, Cabrera, and Mozambique were provided by various surveys conducted by the Spanish Institute of Oceanography, and samples from the Gulf of Lion and the Ionian Sea were provided by the Mediterranean International Bottom Trawl Survey (MEDITS08). The remaining samples were obtained by local fishermen and collected on the date of capture. Details of the samples are in Fernández *et al*.^2^. A piece of muscle from each of the 494 collected individuals was stored in 95% ethanol for later DNA analysis.

### DNA extraction and microsatellite genotyping

We performed genomic DNA extraction following the standard phenol-chloroform procedure, and we carried out genotyping for 12 microsatellites developed for *A. antennatus*: Aa123, Aa138, Aa496b, Aa667, Aa681, Aa751, Aa818, Aa956, Aa1061, Aa1195, Aa1255, and Aa1444, following Heras *et al*.^13^. These molecular markers were distributed in three multiplex PCRs (sets of 3–5 loci) and one singleplex PCR for optimal amplification, following standard procedures with an annealing temperature of 50 °C or 60 °C, as described in Planella *et al*.^22^. PCR product sizing was performed with the GeneScan^TM^ 500 LIZ^®^ size standard (Applied Biosystems) on an ABI PRISM^®^ 3130 Genetic Analyzer (Applied Biosystems). Allele scoring was performed with Geneious v. 7.1.9 software^47^.

### Genetic diversity within locations and effective population size

We calculated allele number (*N*a) and richness (Ar) per locus and per population with FSTAT v. 2.9.3.2 software^48^, and computed the number of private alleles (Ap) with GenAlEx v. 6.503 software^49^. We estimated observed and expected heterozygosities (*H*o and *H*e), and inbreeding coefficient *F*_IS_^50^ and tested for linkage and HW equilibria using Genepop v. 4.4.3 software^51^. We assessed test significance using exact tests (Markov chain Monte Carlo method with 10,000 dememorizations and 5,000 iterations) and Fisher’s method for global tests. Significance values were adjusted for multiple comparisons using Bonferroni correction. Micro-Checker software^52^ was employed to identify scoring errors due to large allele drop-out, stuttering or presence of null alleles.

We estimated the contemporary effective population size (*N*_*e*_) for each location using NeEstimator v. 2.1 software^19^ with the Linkage Disequilibrium method under a random mating model, and we determined 95% confidence intervals using the non-parametric jack-knife method. Given the minimum sample size of 38 (Table 1), a critical allele frequency (Pcrit) of 0.01 was used to exclude alleles occurring in a single copy, ensuring that Pcrit >1/2N.

### Genetic differentiation between locations and gene flow

Pairwise *F*_ST_ values^50^ between locations were calculated using Arlequin v. 3.5.1.2 software^53^, with 10,000 permutations to test significance. We used the Bonferroni correction for multiple comparisons.

The FreeNA programme^54^ was used to assess the effect of null alleles when genetic differentiation (*F*_ST_) was inferred. We estimated the frequency of null alleles and computed *F*_ST_ values that were and were not corrected for the presence of null alleles using 1,000 bootstrap replicates.

We conducted a global locus-by-locus AMOVA analysis with Arlequin v. 3.5.1.2 software^53^, computing *F*-statistics derived from hierarchical partitions. Four different a priori grouping scenarios were independently analyzed to test whether geographic and oceanographic discontinuities effectively reduced gene flow across the species distribution (Fig. 1, Table 3): (i) four groups based on previous mtDNA results, with the Strait of Gibraltar and Strait of Sicily acting and the sample from Mozambique Channel isolated from the others^2^; and (ii) additional groupings with modification of the effectiveness of various barriers. Statistical significance of *F*-statistics for each scenario was tested with 10,000 permutations. In such approach, the best grouping scenario maximizes *F*_CT_ by reducing *F*_SC._

We used Populations v. 1.2.32 software^55^ to construct an NJ tree based on Latter’s *F*_ST_ genetic distance^56^. The robustness of the tree was tested using bootstrap analysis with 1,000 replicates. DAPC is a multivariate model-free type of analysis conducted with no assumption of HW or linkage equilibrium^57^. According to Pometti *et al*.^58^, it allows more rapid and accurate inference of *K* populations than do Bayesian model–based clustering methods. We conducted DAPC with adegenet v. 2.1.0^59^ to identify genetic clusters among sampled individuals. We also computed membership probabilities based on the retained discriminant functions, and we used a compoplot function to interpret group memberships in DAPC and to infer admixture between clusters.

IBD was tested with the ISOLDE programme in Genepop v. 4.4.3^51^. We used the Mantel test based on 100,000 permutations and correlated the logarithm of the pairwise geographical distances between locations and pairwise estimates of linearized *F*_ST_ [*F*_ST_/(1 − *F*_ST_)], obtained with FreeNA. The geographical distances between sampling sites were measured as the shortest marine map distance (in kilometers) in Google Earth based on two hypothesised dispersal routes: (i) that connecting Mozambique with Atlantic populations through the Cape of Good Hope and along the Atlantic coast of Africa, and (ii) passage through the Red Sea and Suez Canal, and then through the Mediterranean Sea.

Finally, we generated a network graph representing relative directional migration rates between regions, considering the locations adjoining the oceanographic barriers, with divMigrate-online^60^ (https://popgen.shinyapps.io/divMigrate-online/) using *Nm*^61^ as a measure of genetic distance. Population samples (or groups of samples) are represented by nodes in the networks. In addition, to test whether the gene flow among regions was asymmetrically significant, 1,000 bootstrap replicates were performed (*α* = 0.05).

## Supplementary information

Supplementary Information

## Data Availability

All data are included in the article.
