# Supplementary material for: Genetic structure and population connectivity of the blue and red shrimp Aristeus antennatus
Source: Sci Rep. 2019 Sep 19;9:13531. doi: 10.1038/s41598-019-49958-5 (PMC6753075; doi:10.1038/s41598-019-49958-5)
Supplement: Supplementary file 1 — Supplementary Information [file 41598_2019_49958_MOESM1_ESM.pdf]

Supplementary information for

Genetic structure and population connectivity of the blue and red shrimp *Aristeus antennatus*.

Sandra Heras[1], Laia Planella[1], José-Luis García-Marín[1], Manuel Vera[1,2], María Inés Roldán[1]*

[1] *Laboratori d'Ictiologia Genètica, Universitat de Girona, Girona,* Spain.

[2] Present address: *Departamento de Zoología, Genética y Antropología Física, Campus Lugo, Universidade de Santiago de Compostela, Lugo,* Spain.

*Corresponding author:

E-mail: marina.roldan@udg.edu

This pdf file includes:

Supplementary Table S1
Supplementary Table S2
Supplementary Figure S3
Supplementary Figure S4
Supplementary Table S5

Supplementary Table S1. *P-values* of Hardy Weinberg test per locus and per sample locations. Sample location codes are given in Table 1. Significance after Bonferroni correction indicated in bold ($p < 0.00038$).

	**Fa**	**Alb**	**Al**	**So**	**Ca**	**Pa**	**GL**	**Ge**	**Po**	**IS**	**Moz**
**Aa123**	0.0035	0.6226	0.7129	0.8657	0.4958	0.0428	0.0993	0.1135	0.7090	0.0085	**0.0000**
**Aa138**	0.0389	**0.0000**	0.0371	0.4831	0.1959	0.0259	0.0052	0.4886	0.0033	**0.0000**	**0.0000**
**Aa496b**	1.0000	0.2476	0.5003	1.0000	1.0000	1.0000	1.0000	1.0000	1.0000	1.0000	0.0011
**Aa681**	0.0006	**0.0000**	0.0584	0.0030	0.2031	0.0021	**0.0000**	**0.0000**	**0.0000**	0.0040	0.2697
**Aa751**	1.0000	0.0007	0.1753	**0.0001**	0.0012	0.0009	**0.0001**	**0.0000**	0.0073	0.0902	0.9321
**Aa667**	0.0801	**0.0000**	0.1196	0.0153	0.0311	**0.0000**	0.3598	0.0324	0.0106	0.0068	0.1796
**Aa818**	**0.0000**	**0.0000**	**0.0000**	0.0301	**0.0000**	**0.0000**	**0.0000**	**0.0000**	**0.0000**	0.0017	**0.0000**
**Aa956**	0.0859	0.8473	0.5355	**0.0000**	0.5412	0.1277	0.0178	0.4228	0.0318	0.0030	0.0021
**Aa1061**	0.0080	**0.0000**	0.0005	**0.0000**	0.0064	**0.0000**	**0.0000**	**0.0001**	**0.0001**	**0.0000**	**0.0000**
**Aa1195**	0.3184	0.2780	0.3752	0.1120	0.9531	0.9834	0.9202	0.3547	0.7255	0.3988	0.1285
**Aa1255**	**0.0000**	**0.0000**	**0.0000**	**0.0000**	**0.0000**	**0.0000**	**0.0000**	**0.0000**	**0.0000**	**0.0000**	**0.0000**
**Aa1444**	**0.0000**	**0.0000**	**0.0000**	**0.0000**	**0.0000**	**0.0000**	**0.0000**	**0.0000**	**0.0000**	0.0011	0.0112

Supplementary Table S2. Pairwise F_{ST} values [corrected (above diagonal) and uncorrected (below diagonal) for null alleles]. Location codes are given in Table 1.

	**Fa**	**Alb**	**Al**	**So**	**Ca**	**Pa**	**GL**	**Ge**	**Po**	**IS**	**Moz**
**Fa**	-	0.03325	0.02897	0.04124	0.03385	0.02836	0.02111	0.02092	0.03103	0.01818	0.02693
**Alb**	0.03646	-	0.00178	0.00616	0.00135	0.00376	0.00163	0.00000	0.00032	0.00801	0.03754
**Al**	0.03134	0.00097	-	0.00889	0.00000	0.00187	0.00274	0.00310	0.00311	0.01129	0.03625
**So**	0.03860	0.00582	0.00687	-	0.00141	0.00861	0.00153	0.00349	0.01036	0.01153	0.02741
**Ca**	0.03179	0.00060	0.00000	0.00000	-	0.00212	0.00000	0.00000	0.00218	0.01217	0.02893
**Pa**	0.03209	0.00527	0.00109	0.00548	0.00000	-	0.00000	0.00480	0.00362	0.01715	0.03749
**GL**	0.02176	0.00089	0.00199	0.00132	0.00000	0.00000	-	0.00000	0.00449	0.00525	0.02764
**Ge**	0.01964	0.00000	0.00136	0.00158	0.00000	0.00232	0.00000	-	0.00195	0.00241	0.02762
**Po**	0.03153	0.00039	0.00128	0.00946	0.00000	0.00292	0.00223	0.00000	-	0.01858	0.04122
**IS**	0.02049	0.00922	0.01285	0.01200	0.01246	0.01846	0.00585	0.00234	0.02117	-	0.02958
**Moz**	0.02566	0.04090	0.03609	0.02646	0.02825	0.04067	0.03017	0.02739	0.04398	0.03273	-

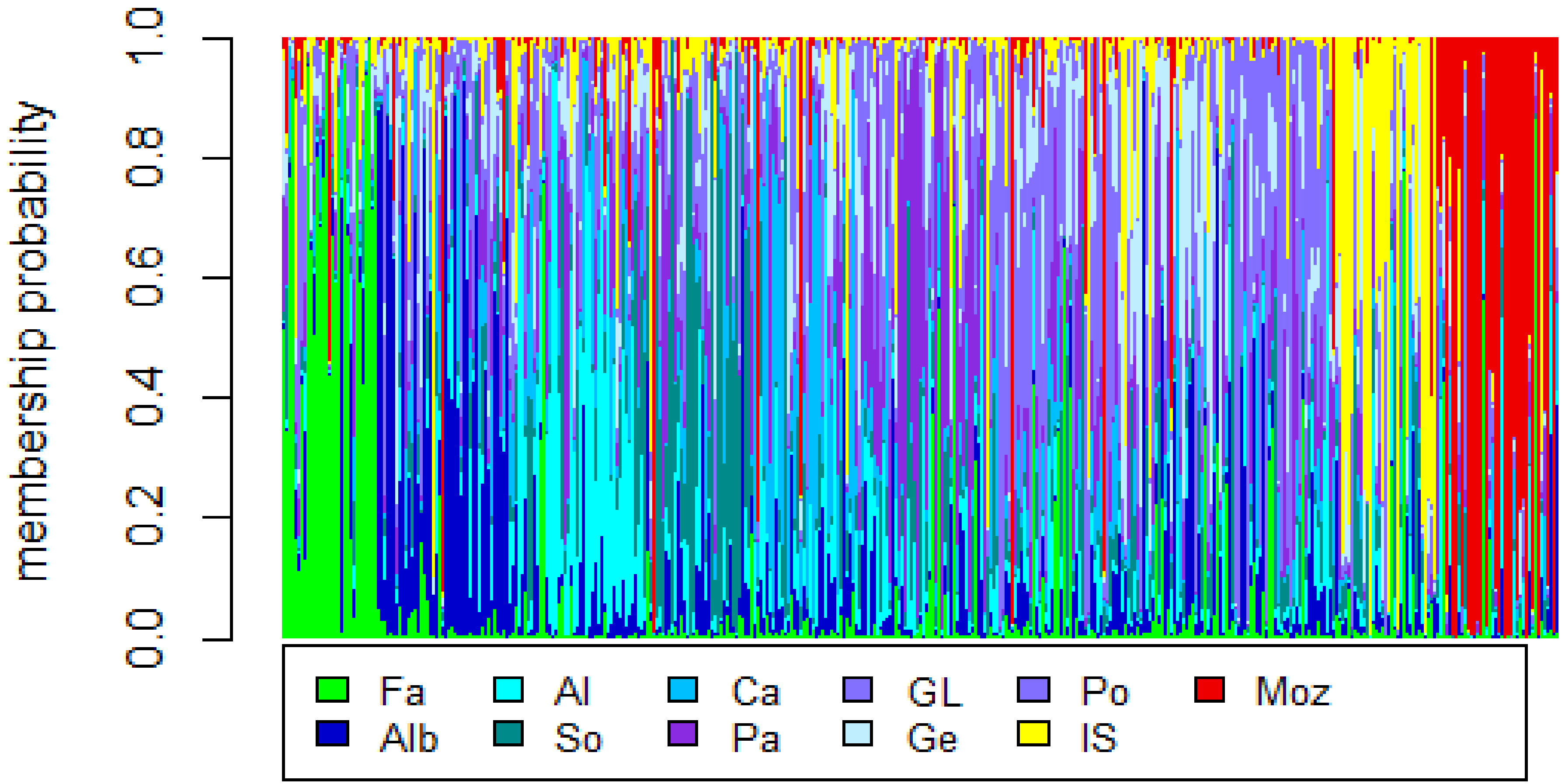

Supplementary Figure S3. Barplot showing the probabilities of assignment of individuals to different locations based on discriminant analysis of principal components functions. Individuals are represented by vertical lines and the proportion of probability of membership to different locations are indicated by different colours. Location codes are given in Table 1.

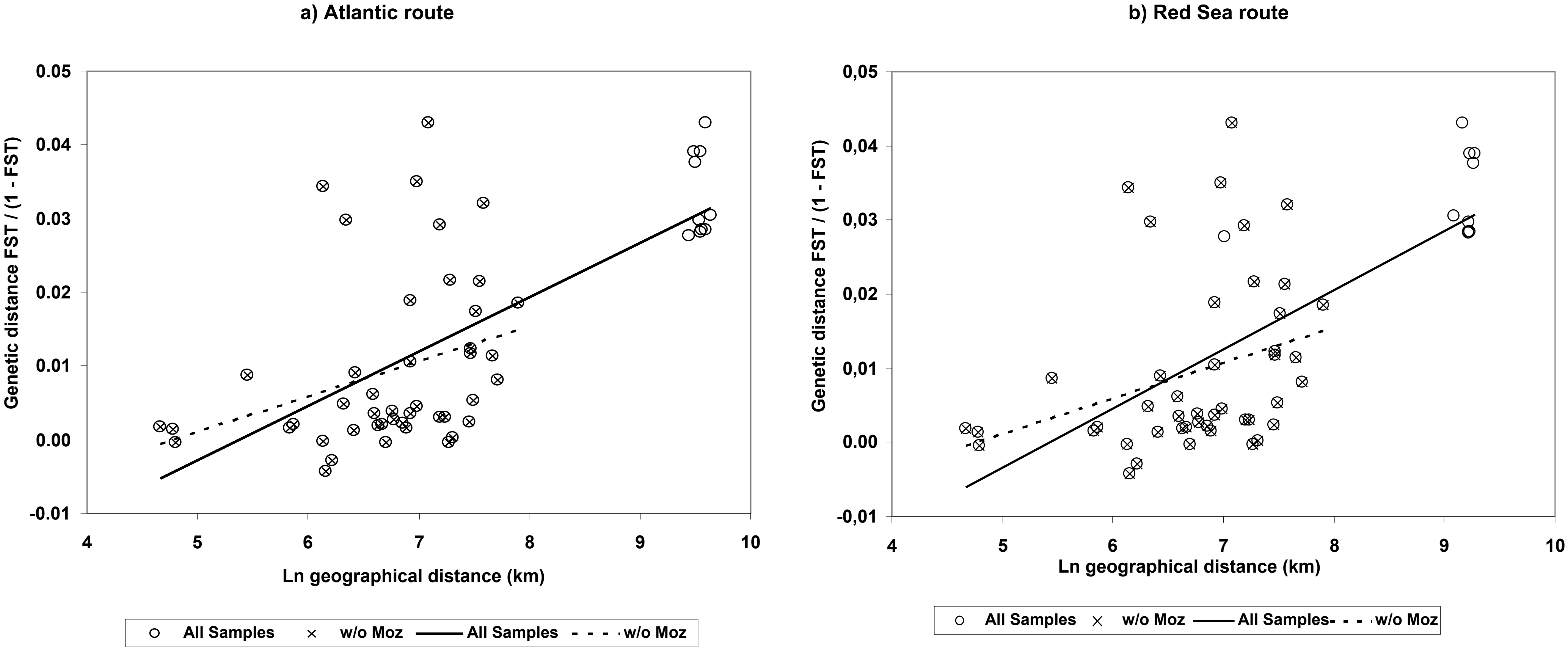

Supplementary Figure S4. Isolation by distance analysis. The genetic distance between pairs of samples (y axis) was plotted against the geographical distance between pairs of samples (x axis). Comparisons involving all samples (open circles) explore connectivity with Mozambique population based on two hypothesised routes **(a)** over the Atlantic route ($r = 0.6757$, $p = 0.00842$) and **(b)** over the Red Sea route ($r = 0.6502$, $p = 0.00783$). Solid lines represent the regression including all samples and dotted lines represent that excluding the Mozambique sample.

Supplementary Table S5. Raw Data Set.

Name	POP	Aa138A	Aa138B	Aa1255A	Aa1255B	Aa956A	Aa956B	Aa496A	Aa496B	Aa123A	Aa123B	Aa681A	Aa681B	Aa667A	Aa667B	Aa1444A	Aa1444B	Aa751A	Aa751B	Aa818A	Aa818B	Aa1061A	Aa1061B	Aa1195A	Aa1195B
Fa483	1	213	223	137	137	213	213	412	412	429	429	236	322	256	256	182	184	230	230	175	175	191	207	195	198
Fa484	1	223	231	155	155	201	213	412	412	427	435	238	240	253	256	188	192	230	230	175	175	207	207	198	201
Fa485	1	217	219	137	137	209	209	412	412	427	429	238	238	250	256	182	192	230	230	175	175	195	207	195	201
Fa486	1	211	211	163	163	213	213	412	412	429	431	238	238	253	253	194	194	230	235	170	170	197	207	198	198
Fa487	1	205	217	125	137	213	213	412	412	427	427	230	258	250	253	182	182	230	230	160	175	191	191	195	198
Fa488	1	201	217	139	171	201	209	412	412	427	427	230	230	256	256	182	192	230	230	170	170	191	191	195	198
Fa489	1	217	223	125	137	205	213	412	412	431	431	240	244	250	256	182	182	230	230	190	190	207	207	201	204
Fa490	1	201	213	137	137	213	213	412	418	427	427	258	258	250	256	192	192	230	230	170	170	207	207	195	201
Fa491	1	199	213	137	139	209	209	412	412	427	427	238	276	256	256	182	198	230	230	175	175	197	207	195	198
Fa492	1	213	213	125	141	205	213	412	412	427	427	236	236	253	256	178	182	230	230	170	170	207	207	198	201
Fa493	1	187	213	139	153	213	213	412	412	427	429	238	270	244	256			230	230	170	170	189	189	201	204
Fa494	1	213	213	137	137	205	213	412	412	427	429	248	322	250	253	186	194	230	230	175	175	207	207	195	198
Fa495	1	211	213	137	137	205	209	412	412	427	431	264	264	250	250	182	182	230	230	170	185	207	207	195	195
Fa496	1	213	217	163	163	205	209	412	412	427	427	236	236	253	253	194	194	230	230	170	175	191	191	195	201
Fa497	1	199	219	137	137	209	209	412	412	427	427	236	238	253	253	186	186	230	230	175	175	191	207	195	195
Fa498	1	213	217	137	153	213	213	412	412	427	427	238	270	250	250	184	184	230	230	165	175	143	207	198	198
Fa499	1	201	213	137	137	205	213	412	412	427	427	260	264	250	256	194	198	230	230	170	175	197	197	195	198
Fa500	1	205	215	137	137	205	209	412	412	427	427	258	258	250	250	186	194	230	230	165	165	207	207	195	198
Fa501	1	205	227	125	125	213	213	412	412	425	427	228	322	244	250	182	182	225	230	170	175	207	207	201	201
Fa502	1	217	221	137	159	213	213	412	412	429	429	224	224	250	256	182	182	230	235	175	175	197	197	195	198
Fa503	1	207	213	137	161	213	213	412	412	429	431	240	248	250	256	194	194	230	230	170	170	191	207	195	198
Fa504	1	221	227	137	139	213	217	412	412	427	427	236	258	253	256	194	194	225	230	170	190	191	207	198	201
Fa505	1	231	231	133	133	205	213	412	412	427	427	240	240	250	256	182	182	230	230	170	170	193	207	195	198
Fa506	1	187	209	137	137	201	213	412	418	427	431	230	240	250	256	182	182	230	230	170	170	191	191	195	195
Fa507	1	201	201	165	165	205	213	412	412	425	429	264	264	247	256	182	184	230	230	160	160	197	207	195	201
Fa508	1	205	213	137	137	213	213	412	412	427	427	236	240	253	256	182	182	230	230	165	175	193	207	195	198
Fa509	1	201	201	135	149	209	213	412	412	427	427	236	236	250	250	186	186	230	230	165	185	207	207	198	201
Fa510	1	207	215	137	141	193	205	412	412	427	427	236	238	250	256	194	194	230	230	165	165	207	207	195	195
Fa511	1	217	247	125	125	209	213	412	412	429	431	238	264	250	250	182	182	230	230	165	165	207	207	195	198
Fa512	1	217	231	137	141	209	213	412	412	427	429	238	264	250	250	182	182	230	230	175	175	207	207	198	201
Fa513	1	207	207	143	143	213	213	412	412	427	427	236	236	256	256	194	194	230	230	175	175	207	207	198	201
Fa514	1	187	199	139	141	213	213	412	412	429	429	248	258	256	256	182	182	225	230	170	170	191	207	195	198
Fa515	1	207	223	125	137	205	213	412	412	427	427	236	264	253	256	182	194	225	230	185	185	191	195	198	198
Fa516	1	211	211	135	135	209	213	412	418	429	429	258	260	253	253	182	186	230	230	170	175	195	195	195	204
Fa517	1	211	211	137	137	209	209	412	412	427	429	264	264	253	253	186	186	230	230	175	175	191	197	201	201
Fa518	1	201	223	125	125	213	213	412	412	429	431	230	236	250	250	186	194	230	235	175	175	207	207	198	201
Fa519	1	201	217	137	137	209	209	412	412	427	427	236	264	250	256	194	194	230	230	175	175	207	207	198	201
Fa520	1	207	221	135	135	205	209	412	412	431	431	240	240	253	256	182	182	230	230	165	165	197	207	195	195
Ab665	2	191	219	125	135	205	205	412	412	427	429	240	240	250	253	182	182	230	230	170	170	197	209	201	201
Ab666	2	213	213	137	163	209	213	412	412	429	431	236	236	256	256	186	194	230	230	170	170	191	207	195	201
Ab667	2	201	213	137	139	213	213	412	412	427	431	230	268	250	256	182	198	230	230	165	170	195	209	198	201
Ab668	2	203	203	135	163	209	213	412	412	431	431	238	250	256	256	186	194	230	230	170	170	191	191	195	195
Ab669	2	219	219	163	163	205	213	412	412	431	431	264	322	250	250	192	192	230	230	170	170	191	191	195	195
Ab670	2	195	215	159	159	213	217	412	412	429	429	240	240	250	253	182	182	230	230	170	175	197	207	198	201
Ab671	2	219	219	137	137	213	213	412	412	431	431	236	264	253	253	182	184	230	230	175	175	207	207	198	201
Ab672	2	187	205	137	137	205	209	412	412	427	431	238	264	250	256	182	192	230	230	165	165	143	191	195	195
Ab673	2	207	223	137	139	205	209	412	412	427	427	248	322	241	253	182	194	230	230	170	170	191	191	195	198
Ab674	2	207	207	155	155	201	205	412	412	429	431	236	236	253	253	182	192	230	230	170	170	197	197	195	201
Ab675	2	213	213	125	141	201	209	412	412	429	431	236	264	253	253	182	182	230	230	180	180	191	191	195	195
Ab676	2	201	217	125	137	205	213	412	412	427	431	264	264	256	256	182	182	230	230	170	170	207	207	195	198
Ab677	2	211	217	137	137	201	209	412	412	429	431	236	264	256	256	182	194	230	230	175	175	207	207	195	195
Ab678	2	203	207	137	141	201	205	412	412	427	431	236	236	250	253	186	186	235	235	185	185	143	191	195	195
Ab679	2	217	231	125	125	201	209	412	412	431	431	228	228	256	256	182	182	230	230	170	170	189	197	195	195
Ab680	2	205	217	125	137	201	213	412	412	427	429	240	240	241	253	192	192	225	230	170	170	207	207	198	198
Ab681	2	213	213	159	159	213	213	412	412	431	431	258	258	250	250	182	184	230	230	175	175	191	207	195	201
Ab682	2	187	207	125	125	209	213	412	412	431	431	240	240	253	253	182	182	230	230	175	175	207	207	195	201
Ab683	2	213	231	137	159	201	201	412	412	427	429	230	248	256	256	182	184	230	230	175	175	191	209	198	198
Ab684	2	201	217	139	139	201	213	418	418	431	433	236	240	253	256	182	198	230	230	165	170	207	207	198	201
Ab685	2	213	223	125	125	213	213	412	412	427	431	322	322	256	256	192	192	230	230	170	170	207	207	195	195
Ab686	2	201	201	137	137	213	213	412	418	431	431	234	234	256	256	184	192	230	230	170	170	207	207	195	198
Ab687	2	191	199	137	137	213	213	412	412	431	431	236	236	256	256	182	182	230	230	170	170	207	207	195	195

Ab688	2	217	223	125	137	205	213	412	412	427	431	230	236	250	250	182	182	230	230	170	170	191	207	195	201
Ab689	2	207	223	125	125	205	213	412	412	427	427	238	238	256	256	184	184	235	235	170	170	205	207	195	195
Ab690	2	213	225	143	143	209	213	412	412	429	431	236	240	253	256	182	182	230	230	170	170	195	205	195	195
Ab691	2	205	213	137	137	213	213	412	418	431	433	264	264	253	256	182	182	230	230	170	170	207	207	198	201
Ab692	2	213	217	143	143	209	213	412	412	429	429	236	236	253	253	186	186	230	230	170	170	205	205	195	198
Ab693	2	205	213	137	137	205	213	412	412	425	431	236	266	250	256	184	192	230	230	170	170	191	207	198	198
Ab694	2	187	223	125	137	201	209	412	412	431	431	224	240	250	253	182	182	230	230	170	170	191	207	195	195
Ab695	2	203	203	137	137	205	205	412	412	425	429	236	236	253	253	192	192	225	225	170	170	197	207	195	198
Ab696	2	201	215	125	125	213	213	412	412	431	431	236	236	250	256	192	192	230	230	165	165	207	207	195	195
Ab697	2	225	229	125	137	205	213	412	412	431	431	238	264	253	256	184	196	230	230	170	170	197	197	198	201
Ab698	2	211	231	139	139	209	213	412	418	431	431	264	314	253	256	182	194	230	230	170	170	197	197	195	195
Ab699	2	195	217	141	141	213	213	412	418	429	431	236	260	253	253	182	182	230	230	170	185	197	209	198	198
Ab700	2	205	217	137	137	209	213	412	412	427	431	236	236	253	253	182	182	230	230	170	170	207	207	195	201
Ab701	2	201	205	135	135	193	213	412	412	429	431	224	240	250	256	184	184	230	230	170	170	191	191	195	195
Ab702	2	205	215	125	125	213	213	412	412	429	431	248	248	241	241	184	194	230	235	185	185	191	207	195	198
Ab703	2	187	201	125	125	205	213	412	412	431	431	236	236	250	250	184	184	230	230	190	190	195	207	195	201
Ab704	2	217	225	137	137	205	209	412	412	431	431	230	236	250	250	194	194	230	230	170	170	207	207	195	198
Ab705	2	201	201	133	133	201	213	412	418	427	431	232	236	256	256	182	182	230	230	170	170	191	195	195	198
Ab706	2	203	211	125	125	205	213	412	412	427	431	238	264	256	256	184	192	230	230	170	170	191	191	195	198
Ab707	2	207	227	125	137	213	213	412	412	427	431	236	316	253	256	186	198	230	230	165	165	207	207	195	195
Ab708	2	203	211	137	137	213	213	412	412	429	429	260	264	253	256	182	194	230	230	170	170	207	207	195	195
Ab709	2	213	213	125	137	213	213	412	412	425	429	240	264	253	253	182	194	230	230	170	170	191	191	198	198
Ab710	2	197	213	141	141	201	213	412	412	431	431	236	240	256	256	182	182	230	235	170	175	191	207	198	201
Ab711	2	197	201	143	143	209	213	412	418	427	429	230	264	253	256	194	194	230	230	175	175	197	197	195	195
Ab712	2	219	219	139	139	205	209	412	412	427	431	236	240	256	256	182	194	230	230	170	170	191	191	195	198
Ab713	2	187	219	137	137	201	213	412	412	427	431	236	276	244	256	184	194	230	230	170	175	191	207	195	198
Ab714	2	205	205	125	125	209	213	412	412	431	431	236	264	253	253	186	194	230	230	170	170	209	209	195	195
Ab715	2	207	207	137	137	213	213	412	412	431	431	236	236	256	256	182	182	210	230	165	165	191	195	195	198
Ab716	2	201	215	137	137	205	213	412	412	429	431	236	282	256	256	194	194	230	230	165	175	191	207	195	198
Ab717	2	199	213	135	135	205	205	412	412	427	431	238	322	250	256	180	186	230	230	170	170	191	191	195	195
Al521	3	203	213	137	137	213	213	412	412	429	431	282	282	256	256	182	182	230	230	170	170	207	207	198	198
Al522	3	195	201	125	137	209	209	412	418	427	429	264	264	253	253	182	194	230	230	170	170	195	207	198	201
Al523	3	213	221	125	125	213	213	412	412	431	431	230	236	256	256	182	182	230	230	170	170	143	195	195	195
Al524	3	211	225	137	137	205	209	412	412	427	429	236	264	250	253	182	182	230	230	165	170	207	207	195	198
Al525	3	201	217	139	139	209	213	412	412	427	427	236	264	250	256	194	194	235	235	170	170	191	191	195	198
Al526	3	217	217	133	137	201	209	412	412	427	429	236	236	241	253	192	194	230	235	170	175	207	207	198	198
Al527	3	211	221	137	137	213	213	412	412	427	427	236	238	256	256	182	182	230	230	170	170	207	207	198	198
Al528	3	207	207	133	137	213	213	412	412	427	431	236	236	253	253	184	186	230	230	170	170	191	191	195	198
Al529	3	201	201	125	143	209	213	412	412	427	427	236	236	253	256	182	182	230	230	170	170	207	207	195	195
Al530	3	187	199	125	133	205	213	412	412	427	431	230	236	250	256	182	184	230	230	170	170	193	193	195	195
Al531	3	201	213	137	137	205	213	412	412	427	431	238	266	250	250	186	194	210	230	175	190	207	207	195	198
Al532	3	201	223	137	137	213	213	412	412	429	431	266	266	250	250	184	184	230	230	170	170	191	207	195	195
Al533	3	201	207	125	137	213	213	412	412	429	431	236	264	250	256	182	196	230	235	170	175	207	207	195	195
Al534	3	207	215	137	137	201	213	412	412	427	429	240	248	253	253	188	192	230	230	170	170	189	207	195	201
Al535	3	205	231	125	137	213	213	412	412	429	431	228	276	253	253	196	196	230	230	170	175	197	207	195	201
Al536	3	223	231	125	137	209	213	412	412	425	427	236	282	250	253	182	184	210	230	170	170	195	195	195	195
Al537	3	199	205	125	139	209	213	412	412	427	431	236	236	250	250	182	182	230	230	165	165	197	197	198	201
Al538	3	217	231	141	141	213	213	412	412	427	429	260	264	253	256	182	182	230	235	170	175	191	207	195	198
Al539	3	205	231	155	155	205	213	412	418	431	431	236	260	250	253	182	182	230	230	175	175	191	207	195	195
Al540	3	205	231	141	141	213	213	412	412	431	431	236	264	253	256	200	200	230	235	190	190	189	207	195	198
Al541	3	221	221	139	139	213	213	412	412	429	431	236	320	250	253	192	192	230	230	170	170	191	191	195	198
Al542	3	199	217	137	137	209	213	412	412	429	429	230	230	253	256	182	194	230	230	170	170	191	197	195	201
Al543	3	201	213	137	137	213	213	412	412	427	429	260	322	253	253	184	192	230	230	165	165	143	143	198	201
Al544	3	201	231	137	137	205	209	412	412	429	431	236	236	256	256	194	194	230	230	170	175	191	191	195	195
Al545	3	205	205	135	143	193	205	412	418	429	429	232	238	253	253	182	194	230	230	170	170	191	207	195	195
Al546	3	191	205	135	135	213	213	412	412	425	431	236	268	256	256	182	194	230	230	170	180	195	207	195	198
Al547	3	201	213	137	137	201	209	412	418	431	431	236	236	250	256	184	192	230	230	165	175	193	207	195	195
Al548	3	207	221	137	137	209	213	412	418	429	431	236	248	250	253	182	194	235	235	170	170	207	207	195	195
Al549	3	211	231	143	143	213	213	412	418	429	431	236	236	253	256	182	194	230	230	175	190	205	205	195	201
Al550	3	199	219	125	137	201	205	412	418	431	431	236	238	250	253	182	182	230	230	170	190	191	197	195	195
Al551	3	213	217	139	139	205	209	412	412	427	431	236	236	250	256	184	198	230	235	165	190	209	209	198	198
Al552	3	195	201	125	125	209	213	412	412	429	429	236	236	253	256	182	182	230	230	170	170	207	207	198	198

Al553	3	211	231	125	125	205	213	412	412	427	429	236	236	250	256	184	194	230	230	165	165	195	195	195	195
Al554	3	219	229	137	141	213	213	412	418	431	431	230	236	256	256	182	194	210	230	165	170	207	207	195	195
Al555	3	195	209	137	137	209	213	412	412	427	427	238	238	250	256	182	182	230	230	170	170	207	207	195	198
Al556	3	231	231	139	139	213	213	418	418	429	431	238	264	253	256	182	192	230	230	170	170	207	207	195	195
Al557	3	191	217	137	155	205	213	412	412	429	431	236	320	256	256	184	184	230	230	190	190	191	191	195	201
Al558	3	205	205	125	125	209	213	412	412	431	431	236	322	256	256	182	182	230	230	180	180	143	207	195	195
Al559	3	201	219	137	137	205	213	412	412	429	431	236	322	253	253	194	194	230	230	170	170	195	195	195	201
Al560	3	199	201	137	139	209	213	412	412	427	431	232	248	241	241	182	194	230	230	165	165	191	207	201	201
Al561	3	211	231	125	137	201	213	412	412	427	431	236	236	253	256	192	192	230	230	165	165	195	207	195	198
Al562	3	195	213	133	137	205	213	412	412	431	431	228	236	253	256	184	184	230	235	170	170	207	207	195	195
Al563	3	195	213	125	137	213	213	412	412	429	431	236	236	250	256	182	182	230	235	170	170	191	207	195	198
Al564	3	199	213	125	125	205	213	412	418	429	431	236	242	247	253	184	194	230	230	170	175	207	209	195	198
Al565	3	201	205	137	137	213	213	412	412	431	431	230	236	250	256	184	194	230	230	170	170	207	207	198	198
So798	4	231	231	139	165	213	213	412	412	429	431	236	236	250	250	184	192	230	230	170	175	207	207	195	198
So799	4	201	207	133	141	205	209	412	412	431	431	236	278	250	256	186	194	235	235	170	170	207	207	195	198
So800	4	195	201	133	133	213	213	412	412	431	431	236	236	256	256	184	184	225	230	165	170	191	197	195	201
So801	4	201	231	139	139	213	213	412	412	429	431	236	264	250	253	194	194	230	230	170	170	195	195	195	198
So802	4	199	213	125	137	201	213	412	412	427	431	236	236	256	256	182	192	230	235	175	175	191	207	195	195
So803	4	207	217	139	153	205	213	412	412	431	431	230	238	253	256	182	194	230	230	170	175	197	197	195	198
So804	4	199	213	135	137	213	213	412	412	429	429	236	264	256	256	192	192	230	230	175	175	207	209	201	204
So805	4	201	201	125	125	209	213	412	412	429	431	224	244	250	250	182	182	230	230	170	175	189	207	195	198
So806	4	213	231	171	171	205	213	412	418	427	427	238	288	244	253	182	194	230	235	170	190	191	191	195	195
So807	4	201	201	137	143	213	213	412	412	431	431	238	264	250	256	192	192	235	235	170	170	207	207	195	201
So808	4	205	217	125	137	205	213	412	412	427	431	298	298	253	253	182	182	235	235	165	175	207	207	195	198
So809	4	203	221	137	139	209	209	412	412	431	431	238	238	250	253	182	194	230	230	175	175	191	191	195	198
So810	4	201	201	125	139	209	209	412	412	431	431	264	264	250	250	182	182	230	230	165	165	209	209	195	198
So811	4	217	221	137	137	213	213	412	412	431	431	236	270	256	256	194	194	230	230	170	170	191	191	195	198
So812	4	217	233	125	133	205	213	412	412	429	431	238	248	250	256	182	184	230	230	170	170	207	207	195	198
So813	4	217	231	141	141	213	213	412	412	429	431	230	248	253	256	180	180	230	230	175	175	207	207	195	195
So814	4	195	231	139	143	213	213	412	412	427	427	236	238	256	256	182	182	230	230	170	175	189	189	195	195
So815	4	207	221	137	139	205	213	412	412	431	431	228	228	250	253	184	184	230	230	165	170	191	191	195	198
So816	4	187	211	137	137	213	213	412	412	431	431	236	238	250	253	192	192	230	230	170	175	191	191	198	201
So817	4	205	205	125	137	205	213	412	412	429	431	236	264	250	253	194	194	230	230	165	185	207	207	195	195
So818	4	201	213	125	137	205	213	412	412	427	431	260	264	250	253	194	194	230	235	170	170	195	195	195	198
So819	4	195	231	137	137	205	213	412	412	431	431	238	282	256	256	182	182	230	235	165	175	195	195	195	201
So820	4	199	227	157	157	209	209	412	412	431	431	238	238	250	253	194	194	230	230	175	175	191	207	195	198
So821	4	211	221	125	137	217	217	412	412	429	431	236	238	253	253	182	192	230	230	170	170	191	191	195	198
So823	4	217	221	125	137	209	213	412	412	429	431	236	236	250	253	192	192	230	230	170	170	197	207	198	198
So824	4	199	217	125	141	213	213	412	412	429	431	230	322	241	250	184	194	230	230	170	175	191	191	195	198
So825	4	191	213	125	137	201	201	412	418	427	429	236	260	256	256	182	182	230	230	170	170	191	207	195	195
So826	4	205	213	125	159	201	201	412	412	431	431	264	264	250	256	194	194	230	230	170	170	191	191	195	201
So827	4	213	217	137	137	205	213	412	412	429	431	236	236	250	253	182	182	230	230	170	170	191	207	195	195
So828	4	201	223	155	155	201	213	412	412	431	431	236	236	253	256	182	186	230	235	170	175	207	207	195	201
So829	4	205	225	137	139	205	209	412	418	431	431	248	248	250	253	192	192	235	235	170	175	207	207	195	198
So830	4	201	201	139	139	205	213	412	412	427	429	260	264	253	253	182	192	230	235	170	170	191	207	195	198
So831	4	217	231	125	133	209	213	412	412	425	431	236	258	253	256	180	198	230	235	165	165	191	191	195	198
So832	4	199	231	125	125	213	213	412	412	427	431	230	236	256	256	182	194	230	230	170	170	191	195	198	198
So833	4	207	217	125	125	213	213	412	412	429	429	236	240	250	256	182	194	235	235	175	175	195	207	192	201
So834	4	191	201	137	137	213	213	412	412	427	429	236	236	256	256	182	182	230	235	165	170	191	197	198	198
So835	4	217	217	137	137	209	209	412	412	427	431	236	238	250	250	194	194	230	230	175	175	205	207	195	195
So836	4	205	217	125	135	213	213	412	412	431	431	236	238	250	256	182	182	230	235	170	175	197	197	195	198
So837	4	205	205	135	135	201	209	412	412	431	431	258	258	253	253	182	182	235	235	170	170	191	207	195	201
So838	4	201	217	137	137	205	209	412	412	431	431	264	264	253	253	182	182	230	235	170	175	191	191	198	198
So839	4	201	217	137	137	213	213	412	412	427	431	236	238	253	256	180	198	235	235	170	170	197	207	195	195
So840	4	217	223	125	125	193	217	412	412	427	427	236	264	256	256	182	194	230	230	165	175	191	191	192	195
So841	4	209	213	141	141	205	205	412	412	427	431	236	240	256	256	182	194	230	230	165	170	195	195	198	198
So842	4	201	205	125	125	209	209	412	412	427	431	236	236	256	256	182	192	230	230	170	170	191	195	195	198
So843	4	213	249	137	137	201	213	412	412	431	433	238	238	253	253	182	182	235	235	170	170	207	207	195	195
So844	4	201	203	137	139	201	209	412	412	427	431	240	240	256	256	188	192	225	225	175	185	207	207	195	201
So845	4	211	231	149	149	209	213	412	412	431	431	228	236	253	256	184	194	235	235	170	170	197	207	195	195
Ca758	5	195	207	159	163	205	213	412	412	431	431	236	236	250	256	182	184	230	235	190	190	191	191	195	195
Ca759	5	217	217	137	137	201	209	412	412	427	427	236	236	244	244	182	182	230	230	170	185	195	207	195	195

Ca760	5	199	205	125	139	205	205	412	412	425	431	236	264	253	256	182	194	210	230	170	170	191	195	198	201
Ca761	5	207	207	137	137	209	213	412	412	431	431	282	282	250	250	182	182	230	235	170	170	191	207	195	198
Ca762	5	205	217	163	163	201	213	412	412	429	429	236	236	250	256	184	184	230	230	165	170	207	207	195	198
Ca763	5	201	219	137	139	209	213	412	412	427	429	236	238	250	253	194	194	230	230	170	175	207	207	195	198
Ca764	5	213	229	137	137	205	213	412	412	427	431	236	236	250	256	182	182	230	230	170	180	207	207	195	201
Ca765	5	203	217	137	139	205	213	412	412	429	431	236	236	250	250	182	192	230	230	170	175	143	191	195	201
Ca766	5	187	211	137	141	213	213	412	412	429	431	236	236	253	256	182	182	230	230	175	190	191	207	195	195
Ca767	5	199	211	135	135	213	213	412	412	429	431	238	242	253	256	194	194	230	230	170	170	191	191	195	195
Ca768	5	217	217	137	137	205	213	412	418	427	429	236	240	256	256	192	194	230	235	170	170	191	207	198	198
Ca769	5	201	231	125	139	201	213	412	412	431	431	236	264	250	253	192	192	235	235	175	190	191	197	198	198
Ca770	5	213	217	125	125	205	213	412	418	431	431	238	266	256	256	182	182	235	235	170	170	191	207	195	201
Ca771	5	205	219	139	139	205	213	412	412	429	431	258	258	256	256	182	194	230	235	170	170	143	191	195	198
Ca772	5	213	217	125	125	213	213	412	412	427	429	236	264	253	256	182	186	230	230	165	170	191	191	195	198
Ca773	5	195	211	137	137	213	213	412	418	431	431	240	258	250	256	182	182	230	230	170	170	207	207	195	198
Ca774	5	205	231	139	139	205	205	412	412	429	431	228	236	256	256	182	182	230	230	170	170	191	195	198	198
Ca775	5	213	213	137	137	213	213	412	412	431	431	236	242	253	256	182	182	235	235	160	175	195	207	198	198
Ca776	5	217	221	137	137	205	213	412	418	431	431	236	236	250	253	184	184	230	235	165	170	191	207	195	198
Ca777	5	201	205	125	137	205	213	412	412	427	431	264	264	250	250	194	194	230	230	170	170	191	207	195	198
Ca778	5	217	235	165	165	209	213	412	418	431	431	268	292	250	256	184	192	230	230	170	170	191	191	195	201
Ca779	5	203	211	125	125	209	209	412	418	427	427	236	294	241	253	188	188	230	230	170	170	197	207	195	198
Ca780	5	213	213	153	153	205	213	412	412	427	429	236	236	250	250	182	182	230	235	190	190	207	207	195	198
Ca781	5	191	201	137	137	201	213	412	418	429	431	236	236	250	253	182	194	230	230	165	175	207	207	195	198
Ca782	5	201	217	137	141	201	213	412	412	427	429	236	236	250	256	192	192	235	235	165	170	191	191	198	198
Ca783	5	203	217	137	137	213	213	412	412	431	431	236	264	250	256	182	194	230	230	175	175	191	197	195	195
Ca784	5	201	201	137	143	201	213	412	412	429	429	236	264	256	256	182	182	230	230	165	185	207	207	195	195
Ca785	5	213	231	125	125	209	213	412	412	427	431	260	264	256	256	184	184	230	235	170	170	143	143	195	195
Ca786	5	203	223	137	137	213	213	412	412	429	431	238	258	250	253	182	182	230	230	170	170	191	205	195	195
Ca787	5	201	207	135	137	205	213	412	412	427	429	236	260	253	256	182	184	230	230	170	170	191	207	195	198
Ca788	5	195	213	125	135	213	213	412	412	427	431	230	236	253	256	182	194	230	230	170	175	207	207	195	195
Ca789	5	203	219	137	137	205	213	412	418	429	431	236	264	253	253	194	198	230	230	170	175	207	207	198	198
Ca790	5	199	211	125	137	213	213	412	412	427	431	236	240	253	253	184	194	230	230	180	180	191	207	198	201
Ca791	5	201	247	137	137	213	213	412	412	431	431	260	320	250	250	182	184	230	235	175	175	197	197	198	201
Ca792	5	191	205	125	125	201	205	412	412	431	431	230	236	253	253	182	184	230	230	170	170	191	191	195	195
Ca793	5	187	187	137	155	205	213	412	412	429	431	236	238	253	256	194	194	230	230	165	175	195	195	195	201
Ca794	5	207	223	133	137	201	213	412	412	429	431	236	240	250	250	194	194	230	230	170	170	207	207	195	198
Ca795	5	213	217	137	139	209	213	412	418	427	429	232	264	253	253	194	194	225	225	170	170	197	197	195	198
Ca796	5	207	217	137	163	213	213	412	412	427	429	236	248	253	253	182	182	230	230	180	180	191	191	198	201
Ca797	5	201	207	133	133	213	213	412	412	427	431	236	238	253	253	182	182	230	230	165	170	207	207	195	195
Pa1104	6	201	223	137	139	193	213	412	412	429	431	236	260	253	253	182	182	225	235	165	165	191	197	195	201
Pa1105	6	213	217	125	137	213	213	412	412	431	431	236	236	253	256	182	184	230	230	170	170	191	191	198	198
Pa1106	6	201	213	125	125	201	209	412	412	431	431	236	264	250	250	184	184	230	230	170	190	191	197	198	201
Pa1107	6	213	221	137	155	213	213	412	418	427	429	236	270	253	253	182	194	230	230	180	180	195	195	195	198
Pa1108	6	199	219	125	125	213	213	412	412	431	431	236	238	256	256	182	182	230	230	170	170	191	207	195	195
Pa1110	6	205	219			213	213	412	412	427	431	236	264	253	253	194	194	230	230	170	170	191	191	195	195
Pa1111	6	213	223	125	139	209	213	412	412	427	429	236	236	256	256	182	192	230	230	170	170	191	191	195	201
Pa1112	6	205	217	139	139	209	213	412	412	427	429	236	236	250	250	182	184	230	230	190	190	191	207	195	201
Pa1113	6	201	213	133	133	205	209	412	412	427	431	238	238	250	250	192	202	230	230	170	170	207	207	195	198
Pa1114	6	205	219	125	133	213	213	412	412	427	427	236	236	253	253	182	182	230	230	165	170	207	207	198	198
Pa1116	6	201	201			209	213	412	412	431	431	236	264	244	256	182	182	230	230	170	170	207	207	195	201
Pa1117	6	201	217	135	137	213	213	412	412	429	429	236	236	250	253			230	230	170	170	191	195	195	198
Pa1118	6	201	227	137	137	213	213	412	412	431	431	236	240	256	256	182	184	225	225	170	170	143	191	195	198
Pa1119	6	199	219	125	137	209	213	412	412	431	431	240	240	253	253	182	182	230	230	170	170	207	207	195	198
Pa1120	6	207	213	133	137	213	213	412	412	427	429	236	240	256	256	182	194	230	230	190	190	207	207	198	201
Pa1121	6	217	231	125	139	205	209	412	412	427	427	236	314	250	256	184	188	230	230	170	170	191	207	195	195
Pa1122	6	213	221			213	213	412	412	431	431	236	236	256	256	182	182	230	230	170	170	191	207	195	195
Pa1123	6	201	201	137	137	213	213	412	412	431	431	236	240	253	253	184	194	230	235	175	175	207	207	195	195
Pa1124	6	203	243	155	155	209	213	412	412	427	431	238	238	250	256	182	192	230	230	170	170	191	191	195	198
Pa1125	6	221	231	137	137	201	209	412	412	431	431	236	238	253	256	194	194	230	230			207	207	195	195
Pa1126	6	201	227	125	125	213	213	412	412	431	431	236	236	250	253	182	194	230	235	170	170	195	195	195	195
Pa1127	6	221	221	125	125	209	209	412	412	429	431	236	236	250	250	192	194	230	230	170	170	191	207	195	195
Pa1128	6	221	221	139	139	205	209	412	412	427	431	236	236	250	256	182	182	230	230	175	175	189	189	195	195
Pa1129	6	211	217	139	139	213	213	412	412	427	427			250	253	192	194	230	235	170	180	189	207	198	198

Pa1130	6	201	217	141	141	213	213	412	412	431	431	238	238	250	250	182	182	230	230	165	175	191	191	195	201
Pa1132	6	201	213	137	155	213	213	412	412	429	429	320	320	250	250	194	194	230	230	170	170	195	195	195	195
Pa1133	6	201	203	137	137	209	213	412	412	427	429	224	236	256	256	180	184	230	230	175	175	191	191	198	201
Pa1134	6	213	231	137	137	205	209	412	412	427	429	230	236	250	256	182	192	230	230	165	170	191	207	195	198
Pa1136	6	195	211	137	137	205	213	412	412	427	431	270	270	256	256	182	182	230	230	170	175	197	207	195	201
Pa1137	6	191	197	139	139	213	213	412	412	425	431	236	260	250	250	194	194	230	230	175	175	191	191	195	201
Pa1138	6	213	217	123	123	213	213	412	418	431	431	236	264	253	256	182	194	230	230	180	180	207	207	195	201
Pa1139	6	187	221	155	163	213	213	412	412	427	431	236	236	250	250	182	182	230	230	175	190	207	207	195	198
Pa1141	6	201	219	137	155	209	213	412	412	431	431	236	236	250	253	188	188	230	230	170	170	189	193	195	195
Pa1142	6	201	217	137	137	213	213	412	412	431	433	238	238	250	253	182	182	230	230	165	170	207	207	198	201
Pa1143	6	201	205	137	141	201	213	412	412	429	431	236	236	250	250	184	194	225	235	170	170	189	191	201	201
Pa1145	6	203	203	125	139	201	209	412	412	429	429	230	238	256	256	192	192	230	230	170	170	207	207	195	201
Pa1146	6	199	211	139	139	205	205	412	412	431	431	260	260	250	250	182	184	230	230	175	175	191	191	195	198
Pa1147	6	227	231	137	137	205	213	412	412	427	427	236	262	250	250	182	186	230	230	175	175	191	205	195	198
Pa1148	6	213	213	137	139	213	213	412	418	427	429	236	322	250	250	182	182	230	230	175	175	191	191	195	195
Pa1149	6	197	197	125	125	205	205	412	412	427	431	236	264	256	256	194	194	230	230	165	170	191	191	195	195
Pa1150	6	201	213	125	125	205	213	412	412	431	433	236	236	250	253	194	194	230	230	165	165	205	205	198	201
Pa1151	6	201	211	135	137	205	213	412	418	431	431	238	238	256	256	182	184	230	235	165	165	207	207	195	198
Pa1152	6	213	213	137	139	213	213	412	412	427	431	236	236	250	250	184	184	230	230	170	170	191	191	195	201
Pa1153	6	213	217	125	125	209	213	412	412	429	431	238	240	253	253	188	188	230	235	165	170	207	207	195	198
Pa1154	6	203	213			205	213	412	412	431	431	238	238	250	250	182	182	230	230	175	175	207	207	192	195
Pa1155	6	205	211	137	143	209	213	412	412	427	427	236	236	250	256	182	186	230	230	170	175	191	207	195	195
Pa1156	6	211	227	125	139	201	205	412	412	431	431	236	248	256	256	182	182	230	230	165	170	191	207	195	195
Pa1157	6	231	231	137	137	213	213	412	418	431	431	264	264	256	256	184	184	230	230	170	175	209	209	195	198
Pa1158	6	203	231	125	137	209	213	412	412	427	431	244	244	250	250	184	184	230	230	170	170	207	207	195	195
GL614	7	187	213	137	137	205	213	412	412	431	431	236	236	256	256	182	182	230	230	175	175	191	195	195	195
GL615	7	199	213	139	157	193	217	412	412	427	427	236	236	256	256	182	194	230	230	175	175	207	207	195	198
GL617	7	201	213	137	137	201	213	412	418	429	429	264	264	250	253	182	182	230	230	175	175	207	207	195	198
GL618	7	199	231	139	161	201	213	412	412	431	431	230	236	250	256			230	230	165	165	191	191	192	198
GL619	7	205	217	133	139	205	205	412	412	429	431	236	236	250	250	182	182	230	230	170	170	143	195	195	201
GL620	7	201	213	137	137	209	209	412	412	429	431	236	250	253	256	182	194	230	230	175	175	207	207	195	198
GL621	7	213	213	137	139	209	213	412	412	431	431	236	250	253	253	170	170	230	230	165	170	195	195	195	198
GL622	7	201	205	139	139	209	213	412	412	429	431	236	236	256	256	184	184	230	230	170	190	207	207	195	198
GL623	7	205	217	137	139	201	213	412	412	427	427	236	322	256	256	182	182	230	230	165	175	191	191	195	198
GL624	7	211	231	139	141	201	209	412	412	431	431	268	268	253	256	182	182	230	230	170	170	207	207	195	201
GL625	7	207	213	137	137	201	213	412	412	425	427	264	264	247	256	182	182	235	235	175	175	191	207	195	198
GL626	7	205	227	137	137	205	209	412	412	427	429	288	320	253	256	182	182	230	230	165	175	207	207	195	195
GL627	7	201	217	125	137	209	213	412	412	429	429	264	264	253	256	182	184	230	230	170	175	191	191	195	198
GL628	7	203	217	125	139	213	213	412	412	427	431	236	238	250	253	186	192	230	230	170	170	207	207	195	198
GL629	7	201	201	159	159	205	205	412	412	431	431	240	264	250	256	182	182	230	235	165	175	191	191	195	198
GL630	7	201	215	137	137	209	213	412	412	427	431	236	238	253	253	182	182	230	230	175	175	143	207	195	195
GL631	7	201	211	137	139	205	209	412	412	429	429	236	240	256	256	182	194	230	230	165	165	191	207	195	198
GL632	7	201	213	133	133	201	213	412	412	431	431	286	286	250	250	182	194	230	230	170	170	191	207	195	201
GL633	7	205	217	137	137	209	209	412	412	427	427	228	230	253	253	182	182	230	230	165	165	207	207	195	201
GL634	7	205	213	137	137	201	213	412	412	431	431	238	260	241	250	182	182	230	230	170	170	191	207	195	198
GL635	7	201	213	133	139	209	213	412	412	431	431	236	236	244	253	192	192	230	230	170	175	207	207	195	195
GL636	7	201	201	137	139	213	213	412	412	431	431	320	320	253	256	182	194	230	230	165	170	207	207	195	198
GL637	7	219	219	137	137	205	213	412	412	429	431	258	258	244	256	190	190	230	230	170	170	191	207	195	195
GL638	7	213	213	137	165	201	213	412	412	427	431	236	236	253	253	194	202	230	230	170	170	207	207	198	201
GL639	7	199	199	125	125	201	213	412	412	427	431	236	236	250	256	194	202	230	230	170	170	207	207	198	201
GL640	7	201	201	137	137	205	213	412	412	429	431	236	236	250	256	182	194	230	230	170	175	197	207	195	195
GL641	7	201	201	125	125	213	213	412	412	427	429	224	224	253	256	192	194	230	230	170	170	207	207	195	195
GL642	7	213	221	135	135	213	213	412	412	427	427	236	236	250	250	192	194	230	230	165	170	191	207	195	201
GL643	7	213	213	135	135	213	213	412	418	427	427	236	236	256	256	188	194	225	225	175	175	207	207	198	198
GL644	7	201	205	125	143	213	213	412	412	431	431	236	238	250	256	182	192	230	230	170	170	191	191	195	195
GL645	7	213	213	137	145	209	213	412	412	427	429	264	264	250	250	198	198	230	230	185	185	207	207	195	201
GL646	7	205	217	137	137	213	213	412	412	431	431	238	260	253	256	194	194	230	230	170	170	191	191	198	198
GL647	7	223	223	125	125	209	213	412	412	429	431	238	238	250	253	194	194	230	230	170	170	197	207	198	201
GL648	7	187	213	137	137	213	213	412	412	429	431	236	240	256	256	182	198	225	225	170	170	191	191	198	198
GL649	7	201	217	139	159	205	209	412	412	431	431	236	244	250	253	184	194	230	230	170	170	191	191	195	198
GL650	7	199	217	125	125	213	213	412	412	427	429	232	264	250	256	184	184	230	230	165	170	189	205	201	201
GL651	7	181	205	125	125	205	205	412	412	429	431	236	236	250	250	184	184	230	235	170	170	191	191	195	201

GL652	7	201	211	125	137	209	213	412	412	431	431	238	264	253	256	182	182	230	235	175	175	189	209	195	198
GL653	7	205	213	167	167	201	205	412	412	427	431	240	244	250	256	184	184	230	230	170	170	207	207	198	198
GL654	7	205	217	125	139	213	213	412	412	429	429	226	236	250	253	182	182	230	230	175	175	207	207	195	195
GL655	7	201	231	137	139	205	213	412	412	429	429	238	250	253	256	182	182	230	235	170	170	207	207	198	198
GL656	7	207	213	133	133	213	213	412	412	427	431	236	316	250	256	182	182	230	230	165	170	191	197	195	195
GL657	7	201	201	125	139	209	213	412	412	429	431	240	240	253	256	182	182	230	230	170	180	143	195	198	201
GL658	7	201	211	125	137	213	213	412	418	431	431	264	282	250	250	180	192	230	230	170	180	191	207	195	195
GL659	7	201	207	125	137	205	209	412	412	429	431	236	236	250	253	194	194	230	230	170	170	191	191	198	201
GL660	7	195	219	125	125	205	209	412	412	429	431	224	236	250	250	192	192	235	235	175	175	207	207	195	198
GL661	7	201	223	137	137	201	213	412	412	427	431	250	282	250	250	198	198	230	230	170	170	191	207	195	201
GL662	7	199	217	141	141	205	205	412	412	429	431	240	250	253	253	182	188	230	230	170	175	191	191	195	198
GL663	7	201	201	137	159	213	213	412	412	431	431	230	316	250	256	182	194	230	235	175	175	191	191	195	195
GL664	7	201	207	137	165	201	205	412	412	427	431	236	260	253	253	182	184	230	230	170	170	191	197	195	198
Ge399	8	203	213	125	137	201	205	412	412	427	429	238	238	250	256	182	182	230	230	170	170	191	191	198	198
Ge400	8	201	205	139	143	205	213	412	412	431	431	264	264	250	256	182	182	230	230	165	170	207	207	195	198
Ge401	8	199	231	125	139	205	205	412	412	427	429	236	236	253	256	184	184	235	235	170	175	207	207	195	198
Ge402	8	195	213	125	125	201	213	412	418	427	429	264	322	253	253	182	182	230	230	175	175	191	207	195	201
Ge403	8	207	207	137	139	209	213	412	412	427	429	240	322	253	256	194	194	230	230	170	170	191	191	195	201
Ge404	8	201	219	133	133	213	213	412	412	431	431	236	238	250	256	182	182	230	230	170	170	207	207	198	198
Ge405	8	217	231	135	137	209	213	412	412	431	431	322	322	250	253	182	182	225	235	175	175	207	207	195	195
Ge406	8	221	223	137	137	201	213	412	412	431	431	236	236	250	253	184	194	230	230	170	175	191	191	198	198
Ge407	8	207	213	139	139	213	213	412	412	429	431	238	316	250	256	194	194	230	235	175	175	191	207	195	201
Ge408	8	219	231	137	137	205	213	412	412	427	433	236	322	250	253	182	198	230	230	170	170	207	207	195	198
Ge409	8	207	213	125	139	201	213	412	412	429	431	224	260	253	256	182	194	230	230	165	165	207	207	198	198
Ge410	8	207	219	137	137	213	213	412	418	427	431	240	264	253	256	182	194	230	230	185	185	209	209	195	198
Ge411	8	199	205	137	137	209	209	412	412	427	431	262	270	256	256	192	194	230	230	165	165	191	205	195	195
Ge412	8	201	231	137	137	205	213	412	412	429	431	264	264	256	256	192	194	230	230	170	175	207	207	195	198
Ge413	8	213	213	137	137	213	213	412	412	429	431	230	236	256	256	182	194	230	230	175	175	191	191	195	198
Ge414	8	195	211	133	137	205	213	412	412	431	431	236	236	250	253	182	182	230	230	170	170	191	207	201	201
Ge415	8	213	213	135	139	209	209	412	412	427	431	238	248	250	256	182	182	230	230	170	190	207	207	195	201
Ge416	8	209	213	155	155	209	213	412	412	427	431	236	236	253	256	182	182	225	235	175	175	195	197	195	201
Ge417	8	219	219	125	137	205	209	412	412	429	431	258	258	250	253	184	184	230	230	170	175	197	197	195	198
Ge418	8	201	203	137	137	205	213	412	412	429	431	224	264	250	253	184	192	230	230	170	170	207	207	195	201
Ge419	8	213	227	137	163	201	213	412	412	427	427	236	236	253	256	184	184	230	230	165	170	191	191	195	198
Ge420	8	223	231	137	155	213	213	412	418	429	429	260	260	253	256	182	182	230	230	170	170	191	191	195	201
Ge421	8	201	205	139	139	213	213	412	412	427	429	242	264	250	256	192	192	230	230	165	165	195	195	195	201
Ge422	8	213	225	135	139	209	213	412	412	427	429	240	292	253	253	182	182	230	230	170	170	191	195	195	201
Ge423	8	205	217	135	151	213	213	412	412	429	431	230	236	253	256	182	194	230	230	170	180	143	207	195	198
Ge424	8	201	203	125	125	205	213	412	412	427	429	264	264	241	253	182	188	230	230	175	175	207	207	198	201
Ge425	8	213	221	133	139	205	209	412	412	429	431	238	258	250	256	194	194	230	230	165	165	191	205	195	198
Ge426	8	201	221	125	125	205	213	412	412	429	431	240	258	253	256	184	184	230	230	170	170	143	191	195	195
Ge427	8	215	217	137	137	205	205	412	412	429	431	238	248	253	256	182	194	230	230	170	190	191	207	195	195
Ge428	8	223	231	125	125	209	213	412	412	431	431	240	240	253	256	192	192	230	230	170	170	207	207	198	201
Ge429	8	205	213	159	159	201	213	412	412	427	429	236	236	250	250	186	186	235	235	170	175	207	207	195	198
Ge430	8	201	223	125	125	205	213	412	412	429	431	320	320	253	256	182	182	230	230	170	170	191	207	198	201
Ge431	8	217	223	137	137	213	213	412	412	431	431	236	236	253	256	192	192	230	230	175	175	207	207	195	195
Ge432	8	201	223	133	139	213	213	412	412	429	431	236	264	250	250	192	192	230	230	170	175	191	195	198	198
Ge433	8	191	201	137	137	201	213	412	412	431	431	236	238	253	256	182	182	235	235	170	170	191	191	195	198
Ge434	8	205	219	125	165	213	213	412	418	427	431	258	258	253	256	182	182	230	235	170	170	197	207	195	198
Ge435	8	201	201	125	125	209	209	412	412	429	431	260	282	253	256	194	194	230	230	170	170	191	207	198	201
Ge436	8	201	217	125	153	213	213	412	412	431	431	264	264	253	253	182	184	230	230	165	170	191	191	198	201
Ge437	8	213	213	137	137	205	213	412	412	431	431	236	314	250	250	186	194	230	230	170	170	191	207	195	198
Ge438	8	205	213	139	163	213	217	412	412	429	431	240	240	250	250	186	194	230	230	170	170	191	207	195	198
Ge439	8	203	207	125	137	201	213	412	412	431	431	230	260	253	256	192	192	230	230	170	175	191	207	195	201
Ge440	8	207	217	137	165	213	213	412	412	427	431	236	260	253	256	184	184	230	230	175	175	207	207	195	201
Ge441	8	201	205	137	137	213	213	412	412	429	431	236	236	253	256	184	194	230	230	165	165	189	189	195	198
Ge442	8	211	213	137	153	213	213	412	412	427	427	236	264	253	256	182	182	230	230	165	170	191	207	198	198
Po443	9	223	229	141	141	213	213	412	418	427	431	236	236	253	256	182	182	230	230	165	175	207	207	195	198
Po444	9	217	217	125	125	205	209	412	412	427	429	238	264	253	256	192	194	230	230	165	165	195	195	195	198
Po445	9	205	217	139	139	213	213	412	418	427	427	236	268	250	253	182	182	230	230	170	170	195	195	195	204
Po446	9	201	231	133	137	213	213	412	418	431	431	240	240	256	256	182	192	230	230	170	170	191	197	195	198
Po447	9	217	217	143	143			412	412	429	431	232	240	250	253	184	184	225	225	175	175	207	209	195	195

Po448	9	205	223	159	159	209	213	412	412	429	431	264	264	253	256	192	192	230	230	175	175	191	207	195	195
Po449	9	213	213	137	137	213	213	412	418	427	435	236	240	253	256	182	182	230	230	165	170	207	207	195	195
Po450	9	217	231	137	143	213	213	412	412	431	431	238	262	247	253	182	194	230	230	165	165	191	207	195	195
Po451	9	201	201	137	137	213	213	412	412	427	431	264	264	250	256	182	182	230	230	165	165	207	207	195	201
Po452	9	201	201	137	143	209	209	412	418	427	429	240	282	253	256	186	186	230	230	170	170	191	195	195	198
Po453	9	213	219	137	137	205	213	412	412	431	431	262	262	250	253	182	182	230	230	165	170	191	207	198	204
Po454	9	207	213	125	137	205	205	412	412	429	431	236	236	250	250	182	182	230	230	165	165	191	197	195	198
Po455	9	201	217	137	137	213	213	412	418	429	431	236	240	241	241	186	186	230	230	170	170	179	191	198	201
Po456	9	215	219	137	137	213	213	412	412	429	429	236	322	250	250	186	194	230	230	155	155	179	195	195	195
Po457	9	219	231	125	137	193	193	412	412	427	431	238	268	253	256	194	198	230	230	180	180	191	197	195	195
Po458	9	213	223	137	137	209	209	412	412	427	427	236	320	250	256	182	182	230	230	170	170			192	201
Po459	9	211	217	137	137	213	213	412	418	427	431	242	264	250	253	184	192	230	230	170	170	191	197	195	195
Po460	9	201	211			213	213	412	412	427	431	226	238	253	256	184	184	230	230	175	175	191	197	198	201
Po461	9	201	221	125	153	213	213	412	412	431	431	238	264	250	253	182	192	230	230	170	170	207	207	195	195
Po462	9	195	215	137	137	213	213	412	412	431	431	238	268	256	256	182	194	230	230	180	180	195	207	198	198
Po463	9	217	219	137	137	213	213	412	412	429	431	228	234	253	256	180	196	230	230	175	175	189	207	195	204
Po464	9	211	221	137	137	205	213	412	412	431	431	238	240	250	253	182	182	230	230	170	170	191	191	195	201
Po465	9	213	221	125	125	205	213	412	412	431	431	236	282	253	256	182	182	230	230	175	175	207	207	198	198
Po466	9	199	213	125	133	205	205	412	412	427	429	236	258	256	256	182	184	230	230	170	175	207	207	198	198
Po467	9	205	205	137	137	205	213	412	418	429	431	236	236	253	256	198	198	230	230	175	175	191	191	195	195
Po468	9	219	227	137	137	213	213	412	412	431	431	236	236	250	256	182	194	225	235	170	170	205	207	195	198
Po469	9	211	211	125	137	205	213	412	412	427	431	236	236	250	253	182	194	230	230	170	170	207	207	195	195
Po470	9	205	205	135	137	201	213	412	412	431	431	236	238	250	253	182	182	230	230	175	175	207	207	198	198
Po471	9	201	217	137	137	213	213	412	412	427	431	258	258	253	256	182	182	230	230	170	170	191	207	195	195
Po472	9	207	207			205	213	412	412	431	431	236	264	250	253	182	182	230	230	165	165	191	191	195	198
Po473	9	213	221	137	143	201	213	412	412	427	431	236	236	253	253	182	182	225	230	170	170	191	207	195	201
Po474	9	201	207	125	137	213	213	412	418	431	431	248	248	256	256	182	182	230	230	165	165	191	191	198	201
Po475	9	215	231	125	125	201	213	412	412	429	431	236	236	253	253	182	192	230	230	170	170	207	207	198	198
Po476	9	195	211	139	155	205	213	412	412	427	431	236	236	253	253	182	186	230	230	170	170	191	191	195	198
Po477	9	211	211	125	133	205	213	412	412	427	431	238	322	250	253	194	194	230	230	170	170	143	191	195	198
Po478	9	201	213	139	139	209	213	412	412	431	431	236	264	250	250	192	192	230	230	170	170	191	191	195	198
Po479	9	217	231	139	167	213	213	412	412	431	431	236	236	253	256	182	184	225	230	165	170	207	207	192	195
Po480	9	197	231	143	143	213	213	412	412	427	429	236	264	253	256	192	192	230	230	165	165	209	209	195	198
Po481	9	199	199	137	137	205	213	412	418	427	431	230	266	253	256	182	182	230	230	170	170	191	191	195	198
Po482	9	227	231	125	125	193	213	412	412	431	431	236	236	256	256	182	192	230	230	165	170			198	201
Io718	10	203	213	125	125	213	213	412	412	429	431	236	236	247	256	194	194	235	235	170	170	191	191	195	201
Io719	10	205	217	137	137	205	205	412	412	427	429	240	264	256	256	184	194	230	230	170	170	207	207	195	195
Io720	10	211	231	133	135	209	213	412	412	427	427	236	236	250	253	192	192	230	230	170	170	207	207	195	198
Io721	10	227	231	139	139	205	205	412	412	427	429	236	236	253	256	182	194	230	230	175	175	191	207	195	198
Io722	10	201	207	137	157	205	213	412	412	431	431	230	238	253	256	182	194	230	230	185	190	191	207	195	195
Io723	10	209	213	125	125	209	213	412	412	429	431	236	322	253	256	194	194	230	235	170	175	207	207	198	198
Io724	10	201	217	155	155	209	213	412	412	427	427	264	322	253	256	182	194	230	230	170	175	207	207	198	198
Io725	10	199	217	133	139	213	213	412	412	431	431	236	250	256	256	182	194	230	230	170	175	203	203	195	195
Io726	10	201	201	133	141	209	213	412	412	431	431	236	258	256	256	182	194	230	230	170	170	191	191	198	198
Io727	10	187	205	125	125	213	213	412	412	427	431	264	264	256	256	182	182	230	230	175	190	207	207	198	198
Io728	10	217	221	125	139	201	213	412	412	427	429	262	264	250	256	182	194	230	235	170	170	191	205	195	198
Io729	10	201	201	137	139	205	213	412	412	431	431	236	264	253	253	182	184	230	235	165	170	191	191	198	201
Io730	10	207	207	137	139	213	213	412	412	431	431	238	260	256	256	192	194	230	230	175	190	207	207	195	201
Io731	10	201	201	157	157	213	213	412	412	427	429	228	264	250	256	180	194	230	235	190	190	207	207	195	198
Io732	10	207	221	133	137	205	205	412	412	427	429	322	322	256	256	192	192	230	235	170	170	191	191	195	195
Io733	10	213	219	137	137	209	213	412	412	429	431	236	264	256	256	182	194	230	230	170	170	191	191	195	198
Io734	10	205	221	125	135	209	213	412	412	427	429	236	236	250	256	194	194	230	235	170	170	207	207	195	201
Io735	10	217	231	125	125	201	205	412	412	427	431	236	322	256	256	182	182	230	230	165	165	207	207	195	198
Io736	10	201	201	137	139	213	213	412	412	427	429	236	238	256	256	192	192	230	230	165	170	207	207	195	198
Io737	10	191	221	137	137	213	213	412	412	427	431	264	322	253	256	182	182	230	230	170	175	207	207	198	201
Io738	10	211	231	137	137	205	205	412	412	427	431	250	250	250	256	182	198	230	230	170	170	191	191	195	201
Io739	10	187	187	137	159	213	213	412	412	427	429	248	322	256	256	196	196	230	230	175	175	205	207	195	201
Io740	10	201	213	137	137	205	213	412	418	427	429	236	322	250	250	192	194	230	230	170	170	191	191	195	198
Io741	10	199	199	137	137	209	209	412	412	429	429	230	282	253	253	186	186	230	230	175	175	195	207	195	198
Io742	10	199	201	125	125	201	213	412	412	427	429	238	238	250	250	194	194	230	230	170	175	191	195	195	198
Io743	10	187	195	139	139	201	205	412	412	429	431	250	264	256	256	182	184	230	230	170	175	207	207	195	198
Io744	10	195	213	139	139	205	213	412	412	431	431	236	236	253	253	182	182	230	230	170	175	207	207	195	198

Io745	10	207	223	137	137	213	213	412	412	427	429	238	238	250	250	194	194	230	230	170	175	207	207	195	201
Io746	10	199	223	125	125	205	205	412	412	429	431	236	236	250	253	192	192	230	230	170	170	207	207	195	201
Io747	10	201	213	135	143	205	205	412	412	431	431	240	264	256	256	192	192	230	230	170	170	191	207	201	201
Io748	10	217	217	137	137	213	213	412	412	427	431	282	282	256	256	182	184	230	230	170	175	143	143	195	198
Io749	10	203	207	125	125	213	217	412	412	429	431	242	248	250	253	182	186	230	230	175	175	207	207	195	201
Io750	10	213	231	137	157	201	213	412	418	431	431	236	236	256	256	192	192	230	230	170	185	191	191	198	201
Io751	10	213	213	125	139	201	213	412	412	427	431	260	264	250	253	182	184	230	230	170	170	195	197	195	201
Io752	10	207	207	137	137	193	209	412	412	427	429	230	264	256	256	194	194	235	235	170	170	207	207	195	198
Io753	10	217	219	137	137	205	205	412	412	427	429	230	266	256	256	184	192	230	230	175	175	207	207	195	195
Io754	10	195	213	137	137	205	205	412	412	431	431	230	322	256	256	182	192	230	230	170	170	205	207	198	198
Io755	10	213	213	137	137	205	205	412	412	427	429	236	236	256	256	192	194	230	230	170	175	191	207	198	201
Io756	10	197	213	167	167	213	213	412	412	427	431	230	240	256	256	182	182	230	230	175	175	207	207	195	201
Io757	10	217	219	139	139	205	213	412	412	431	431	230	322	256	256	182	192	230	230	175	175	209	209	195	198
Mz566	11	201	213	125	125	213	213	412	412	431	433	232	238	250	256	182	188	230	235	170	175	207	207	195	201
Mz567	11	191	225	137	137	213	213	412	412	427	429	224	236	244	256	182	182	230	235	170	175	191	195	195	198
Mz568	11	201	217	137	137	213	213	415	415	429	429	238	280	250	256	182	192	230	235	165	165	143	191	195	201
Mz569	11	213	223	125	125	209	213	412	412	423	427	238	300	256	256	194	194	220	230	165	165	191	195	198	201
Mz570	11	197	219	139	167	205	213	412	412	429	431	238	244	250	256	182	192	230	230	165	175	191	197	201	201
Mz571	11	207	219	151	151	205	213	412	412	427	429	236	248	241	253	182	194	230	230	170	170	191	191	198	201
Mz572	11	225	225	137	139	209	213	412	412	427	429	236	286	247	250	182	182	235	235	170	170	207	207	195	198
Mz573	11	201	217	139	151	209	217	412	412	427	427	242	258	247	253	182	194	235	235	170	175	191	207	198	198
Mz574	11	201	201	137	139	213	213	412	412	427	427	262	264	253	256	182	184	230	235	170	170	191	205	195	198
Mz575	11	213	213	143	157	205	213	412	412	427	429	238	282	250	256	184	184	230	235	165	170	207	207	195	195
Mz576	11	201	215	129	129	205	213	412	412	427	427	238	306	250	253	192	192	230	230	165	165	191	195	195	198
Mz577	11	201	201	137	137	205	205	412	412	429	429	238	240	253	256	190	190	230	230	175	175	191	195	195	198
Mz578	11	195	215			213	213	412	412	427	431	232	232	250	256	182	182	230	235	175	190	207	207	201	201
Mz579	11	203	213	125	137	213	213	412	412	427	429	236	242	247	256	188	198	230	230	170	170	191	195	195	198
Mz580	11	201	217	125	139	201	201	412	412	427	431	244	294	256	256	192	192	230	235	175	190	191	191	195	198
Mz581	11	201	205	161	161	213	213	412	412	427	427	244	264	253	256	182	182	235	235	170	175	197	197	195	198
Mz582	11	203	227	143	143	205	205	412	412	427	427	236	238	250	256	182	182	230	235	170	175	191	191	195	195
Mz583	11	211	217	169	169	209	209	406	412	429	429	240	240	250	259	184	184	230	235	190	190	191	207	198	198
Mz584	11	201	213	125	139	205	213	412	412	427	427	236	236	250	256	182	182	230	230	165	165	193	193	195	195
Mz585	11	189	201	143	143	209	209	412	412	427	431	236	242	250	256	182	186	230	230	190	190	143	207	195	198
Mz586	11	203	203	137	137	205	205	412	412	425	425	232	236	241	256	192	198	235	235	175	175	207	207	201	201
Mz587	11	223	227	137	137	209	213	418	418	427	427	238	238	250	256	182	198	230	230	170	170	207	207	198	198
Mz588	11	217	217	139	139	205	205	412	412	427	427	282	296	253	253	182	196	235	235	170	170	191	191	195	195
Mz589	11	217	217	125	137	209	209	412	412	423	423	240	260	241	250	184	184	230	235	170	170	205	205	195	201
Mz590	11	201	233	159	159	209	209	412	412	429	431	238	242	250	256	182	182	230	235	170	170	195	195	195	201
Mz591	11	195	219	135	137	209	209	412	412	429	431	236	264	253	256	182	184	230	235	170	170	195	197	195	201
Mz592	11	201	201	161	161	205	209	412	412	427	429	240	240	250	250	182	192	235	235	165	165	191	191	195	198
Mz593	11	221	221	125	137	209	209	412	412	429	429	242	276	250	256	188	188	230	235	170	170	191	191	195	198
Mz594	11	195	223	137	137	213	213	412	412	427	427	240	240	253	256	192	194	235	235	190	190	207	207	198	201
Mz595	11	195	207			209	213	412	412	429	429	240	242	247	256	182	182	230	230	175	190	191	191	195	201
Mz596	11	201	205	137	137	209	213	412	412	429	429	242	268	253	253	182	194	235	235	170	170	207	207	195	198
Mz597	11	195	217	137	143	213	213	412	412	427	431	264	276	250	253	182	182	235	235	165	170	191	191	195	198
Mz598	11	195	227	153	153	213	213	412	412	427	427	224	240	253	253	184	184	230	235	175	175	195	207	195	198
Mz599	11	195	217	125	139	205	209	412	412	427	431	230	236	244	256	192	196	230	235	175	175	191	197	195	198
Mz600	11	201	213	141	141	205	209	412	412	427	429	236	236	259	259	184	192	225	230	180	195	191	207	195	198
Mz601	11	217	223	161	161	209	213	412	412	427	429	246	272	244	250	182	194	230	235	165	165	207	207	198	198
Mz602	11	209	219	137	159	205	209	412	412	427	431	236	274	250	256	182	182	230	235	170	190	207	207	195	195
Mz603	11	201	217	125	125	201	213	406	412	427	427	222	264	247	253	182	184	230	235	175	175	207	207	195	198
Mz604	11	201	211	139	171	213	213	412	412	427	427	238	238	256	256	182	182	230	230	175	175	207	207	198	201
Mz605	11	213	225	137	159	205	213	412	412	427	427	238	238	244	256	192	192	230	230	180	180	191	191	195	198
Mz606	11	209	223	137	137	205	213	412	412	429	431	228	240	253	253	182	192	230	235	170	170	191	207	195	198
Mz607	11	199	213	125	141	213	213	412	412	427	427	274	322	256	256	182	184	230	235	170	170	207	207	198	198
Mz608	11	231	243	125	125	201	213	412	412	427	431	236	240	250	256	182	192	235	235	180	180	191	207	198	201
Mz609	11	213	225	137	137	209	209	412	412	431	433	228	230	250	250	182	182	230	235	170	170	191	207	198	201
Mz610	11	197	217			205	213	412	418	425	435	236	260	253	256	182	182	230	230	170	170	207	207	195	198
Mz611	11	201	213	125	137	209	213	412	412	427	427	240	260	247	256	182	182	230	235	170	170	197	207	195	198
Mz612	11	219	219	125	125	205	213	412	412	427	429	240	240	250	256	182	192	230	230	170	175	191	195	195	198
Mz613	11	203	213	137	137	201	201	412	412	427	427	240	272	253	256	182	184	230	230	170	170	143	191	198	201
